# A redox-responsive viologen–cavitand nanocarrier for dual-function photodynamic therapy

**DOI:** 10.3762/bjnano.17.78

**Published:** 2026-08-19

**Authors:** Andrey A Maslennikov, Alexey Yu Usanyov, Amir A Shamsutdinov, Anna P Lyubina, Andrey A Parfenov, Alexandra D Voloshina, Irek R Nizameev, Marsil K Kadirov, Rezeda R Fazleeva, Vitaly V Yanilkin, Kseniya A Zhdanova, Natal’ya A Bragina, Albina Y Ziganshina, Igor S Antipin

**Affiliations:** 1 A. E. Arbuzov Institute of Organic and Physical Chemistry, FRC Kazan Scientific Center, Russian Academy of Sciences, Arbuzov str. 8, Kazan 420088, Russian Federation; 2 Institute of Fine Chemical Technology, MIREA – Russian Technological University, Moscow 119454, Russian Federation; 3 Alexander Butlerov Institute of Chemistry, Kazan Federal University, Kremlevskaya st. 18, 420008 Kazan, Russian Federation; 4 Kazan National Research Technical University named after A.N. Tupolev – KAI, 10 Karl Marx Street, Kazan 420111, Russian Federation; 5 Kazan National Research Technological University, 68 Karl Marx Street, Kazan 420015, Russian Federation

**Keywords:** glutathione-triggered release, *meso*-tetrakis(*p*-hydroxyphenyl)porphyrin, photodynamic therapy, redox-responsive nanocarrier, viologen cavitand

## Abstract

Although photodynamic therapy selectively eradicates tumours via light-triggered reactive oxygen species (ROS) generation, conventional photosensitizers suffer from poor solubility and a reliance on short-wavelength light with limited tissue penetration. To address this, we developed a redox-responsive polymeric nanocarrier through free-radical polymerization of a pre-assembled micellar ensemble. This ensemble formed spontaneously in aqueous solution by encapsulating the hydrophobic photosensitizer *meso*-tetrakis(*p*-hydroxyphenyl)porphyrin within a core composed of the disulfide-containing monomer *N*,*N*′-bis(acryloyl)cystamine, while a viologen-functionalized resorcinarene cavitand anchored at the micelle surface, providing colloidal stability. Polymerization yielded core–shell nanoparticles with a glutathione-cleavable, disulfide-rich core and a viologen-decorated shell. In vitro investigations across a broad panel of six malignant and two non-malignant cell lines demonstrated low dark cytotoxicity and acceptable blood compatibility, with hemolysis remaining below 10%. In the dark, the formulation induced a non-lethal, transient cytostatic effect driven by early-stage apoptosis. Crucially, upon 650 nm laser irradiation, the nanocarrier engaged powerful photodynamic activity, triggering a massive transition to late apoptosis and direct cell death, resulting in near-complete tumor cell eradication. ROS generation, subcellular localization, and programmed cell death mechanisms were confirmed by flow cytometry and fluorescence microscopy, establishing the developed redox-responsive system as an effective candidate for targeted photodynamic therapy.

## Introduction

**Figure 1:**
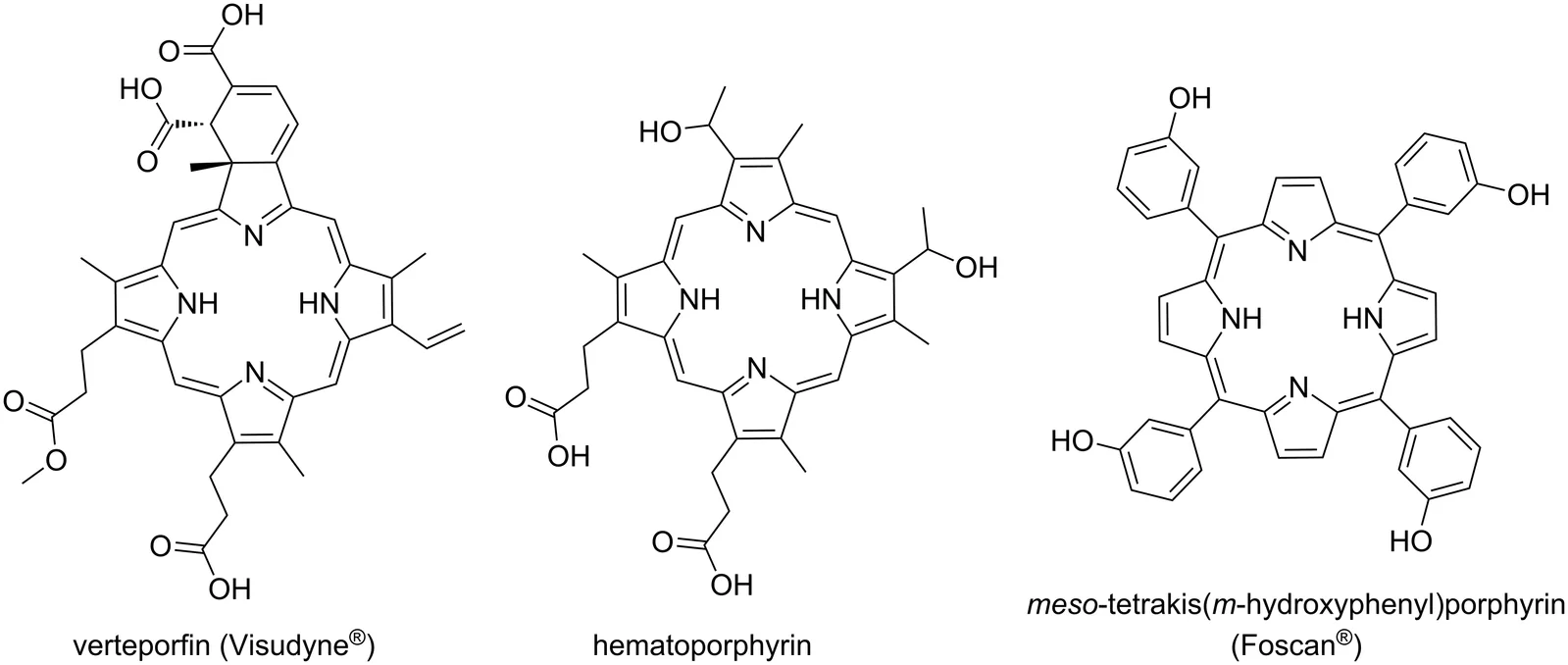
Chemical structures of clinically approved porphyrin-based photosensitizers used in PDT.

Photodynamic therapy (PDT) is a minimally invasive, clinically approved approach widely utilized for cancer treatment, as well as various non-malignant conditions. It relies on three key components, namely, light, molecular oxygen, and a photosensitizer. Upon irradiation, the photosensitizer generates cytotoxic reactive oxygen species (ROS) that selectively destroy target cells [1]. Currently, PDT is actively employed in dermatology for treating skin diseases like actinic keratosis and psoriasis, in ophthalmology for age-related macular degeneration (using Visudyne^®^ (verteporfin)), and in oncology for superficial or endoscopically accessible tumours of the esophagus, lungs, and bladder (using Foscan^®^) [2] (Figure 1). The major advantages of PDT include its high spatial selectivity, low systemic toxicity, and the ability to avoid resistance mechanisms typical of conventional chemotherapy. However, its widespread clinical application is still hampered by major drawbacks, such as limited light penetration depth through thick tissues and the severe hydrophobicity of classic photosensitizers, which causes them to aggregate in physiological media and compromises their photodynamic efficiency [3].

Porphyrins are substances with photodynamic activity. The first of these, hematoporphyrin, was discovered in 1841 by removing iron from dried blood. However, its photodynamic activity and the potential for selective tumour destruction via photodynamic action were discovered only in 1942 by Auler and Banzer. The active development of PDT began in the 1960s [4]. Currently, photosensitizers are divided into three generations [5]: (1) First-generation photosensitizers include hematoporphyrin and its derivatives. They have significant drawbacks, such as low purity, low efficiency in ROS production, and a long elimination period from the body, leading to prolonged skin photosensitivity. Furthermore, they exhibit insufficient tissue penetration due to absorption at shorter wavelengths [6–7]. (2) Second-generation photosensitizers are chemically modified first-generation photosensitizers and their more effective synthetic analogs. They are characterized by higher purity, a higher ROS yield, and an optimal absorption wavelength peak of 650–800 nm, which ensures deeper tissue penetration. These agents cause fewer side effects due to rapid clearance and improved selectivity. However, their main disadvantage is poor water solubility, which limits parenteral administration and requires the design of advanced drug delivery systems [8–9]. The limitations of second-generation photosensitizers subsequently led to the development of third-generation photosensitizers. (3) Third-generation photosensitizers consist of second-generation agents modified with targeting moieties such as antibodies, carbohydrates, amino acids, or peptides; alternatively, they can be encapsulated into delivery systems like liposomes, micelles, or nanoparticles [10]. These vehicles enable the simultaneous delivery of multiple drugs or prodrugs, improve photosensitizer solubility, and significantly extend blood circulation time. To facilitate efficient intracellular uptake via endocytosis, such carriers are designed with minimal diameters typically ranging from 1 to 100 nm. Furthermore, this nanoscale size enhances passive accumulation in tumor tissues due to the enhanced permeability and retention (EPR) effect [11–15], allowing polymer–drug conjugates to achieve intratumoral concentrations 10 to 100 times higher than those obtained with the free drug [16]. However, passive targeting via the EPR effect alone is often insufficient for effective PDT, necessitating strategies that focus on controlled photosensitizer release within the tumor site. While delivery to the tumor extracellular space is sufficient for light-triggered cell membrane destruction, subsequent intracellular internalization further enhances therapeutic efficiency by allowing the simultaneous damage of vital organelles. Ideally, this release is triggered by intrinsic stimuli of the cancer microenvironment, such as low pH, increased enzyme expression, or highly reductive conditions [17–18].

*meso-*Tetrakis(*p*-hydroxyphenyl)porphyrin (THPP) has been extensively studied as an isomer of temoporfin, marketed under the trade name Foscan^®^. However, unlike its meta-isomer, THPP was halted at the preclinical testing stage in favor of Foscan^®^ development [19–20]. Due to the presence of *p*-hydroxyphenyl groups, THPP exhibits an absorption peak at 653 nm. This wavelength falls within the tissue optical window ranging from 650 to 900 nm, which provides optimal penetration through various skin layers and enables the treatment of deeper tumors [21]. Several studies have already demonstrated the successful delivery of THPP using nanoparticles formulated from human serum albumin, poly(ᴅ,ʟ-lactide-*co*-glycolide), and other biocompatible polymers [22–23].

Cancer cells maintain intracellular glutathione (GSH) concentrations 10–1000 times higher than normal tissues. This reductive environment is widely exploited for tumor-selective drug delivery. Disulfide bonds, in particular, undergo rapid cleavage in the presence of GSH. Crosslinkers like *N*,*N*′-bis(acryloyl)cystamine (BAC) thus enable redox-triggered disassembly of polymeric nanocarriers and controlled cargo release [24–25].

Resorcinarene cavitands provide a rigid, bowl-shaped platform for precise functional group display [26–27]. When decorated with methylviologen units, they acquire amphiphilic properties. They spontaneously self-assemble in water around hydrophobic guests forming stable core–shell nanostructures [28–29]. The viologen shell enhances colloidal stability in physiological media [30–31]. It also promotes cellular uptake through electrostatic interactions with negatively charged membranes [32–33]. Moreover, viologens can participate in photoinduced electron transfer, potentially synergizing with porphyrin-based ROS generation to amplify PDT efficacy [34–36].

**Scheme 1:**
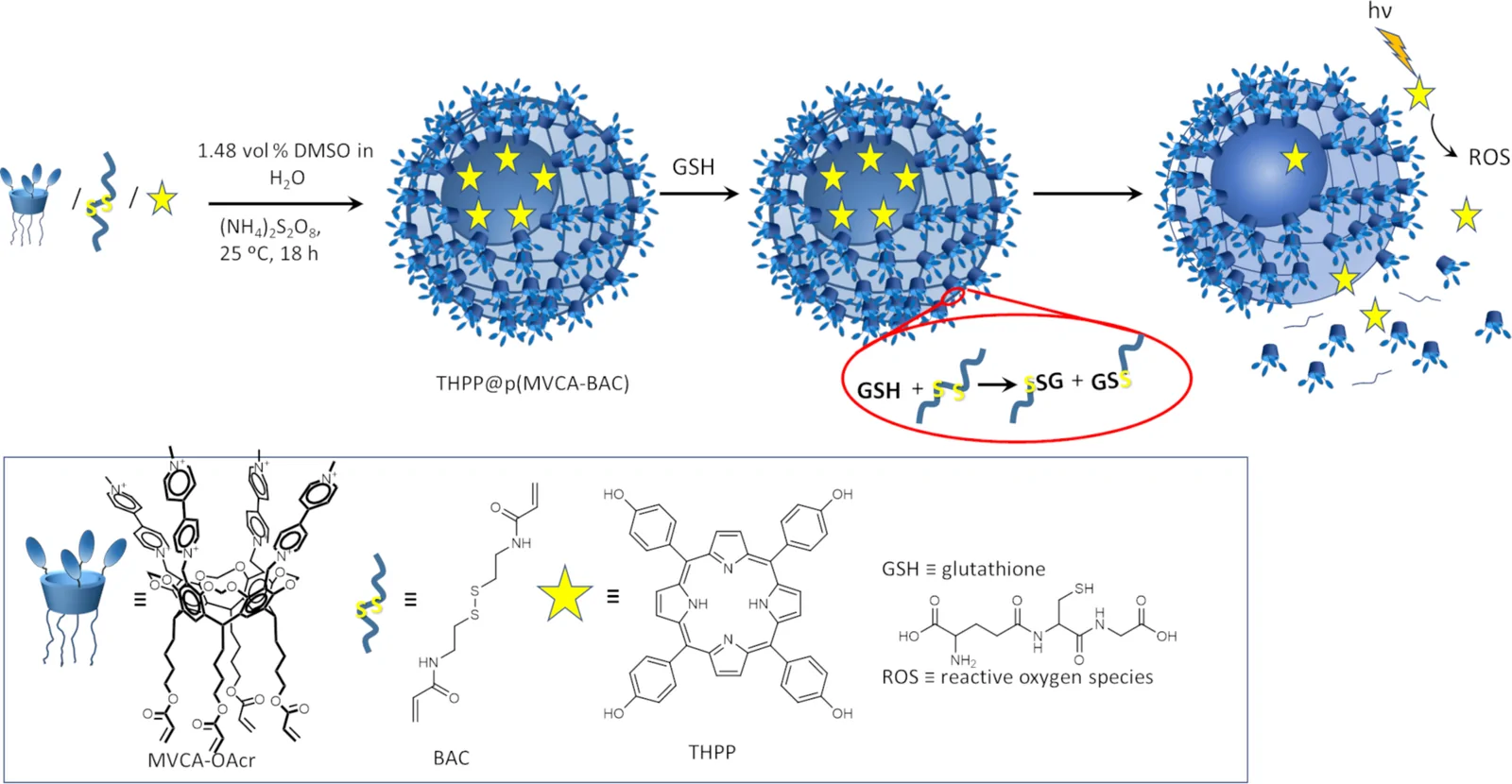
Synthesis of THPP@p(MVCA-BAC) nanoparticles and their GSH-triggered disassembly for ROS generation upon light irradiation.

We developed a redox-responsive nanocarrier for targeted THPP delivery. Monodisperse nanoparticles (THPP@p(MVCA-BAC), see below Scheme 1) were synthesized through self-assembly of viologen functionalized resorcinarene cavitand with acrylate groups on the low rim (MVCA-OAcr, see below Scheme 1) and BAC with THPP, followed by mild radical polymerization. This system achieves high THPP loading, excellent stability, and GSH-triggered release, presenting a rationally designed approach for selective anticancer PDT. Our innovative encapsulation strategy streamlines three functionalities into a single synthetic step to improve upon existing polymer-based methods. A rigid resorcinarene cavitand core provides structural stability and monodispersity without surfactants [26–27]. Peripheral viologen units enhance cellular uptake via positive surface charge and boost reactive oxygen species production through photoinduced electron transfer with THPP [32,34]. In situ disulfide crosslinking prevents premature cargo leakage, enabling efficient GSH-triggered drug release specifically within cancer cells [24]. This one-step multicomponent assembly simplifies previous multistage polymer functionalizations for targeted PDT.

## Results and Discussion

### Synthesis and characterization of THPP@p(MVCA-BAC)

The nanocarrier was constructed via a two-step, surfactant-free strategy, that is, (1) spontaneous self-assembly of molecular components in water, followed by (2) in situ radical copolymerization to lock the structure. The amphiphilic building block, a viologen-functionalized resorcinarene cavitand bearing acrylate groups (MVCA-OAcr), was prepared as previously reported [29]. Upon rapid co-injection of a DMSO solution containing BAC and THPP into an aqueous solution of MVCA-OAcr, instantaneous self-assembly occurred. This assembly was driven by hydrophobic interactions between the BAC/THPP domains and the cavitand aromatic scaffold and hydrophobic tails. Subsequent radical copolymerization produced stable core–shell nanoparticles (THPP@p(MVCA-BAC), Scheme 1).

Monodisperse nanoparticles were obtained by optimizing monomer concentrations, THPP loading, DMSO volume, and polymerization temperature. The narrowest size distribution was achieved using 0.5 mM MVCA-OAcr and 2.5 mM BAC in 15 μL DMSO, which formed well-defined micellar precursors suitable for subsequent polymerization. Higher concentrations and DMSO volumes led to large aggregates or broad size distributions, while lower concentrations encapsulated only small amounts of THPP.

**Figure 2:**
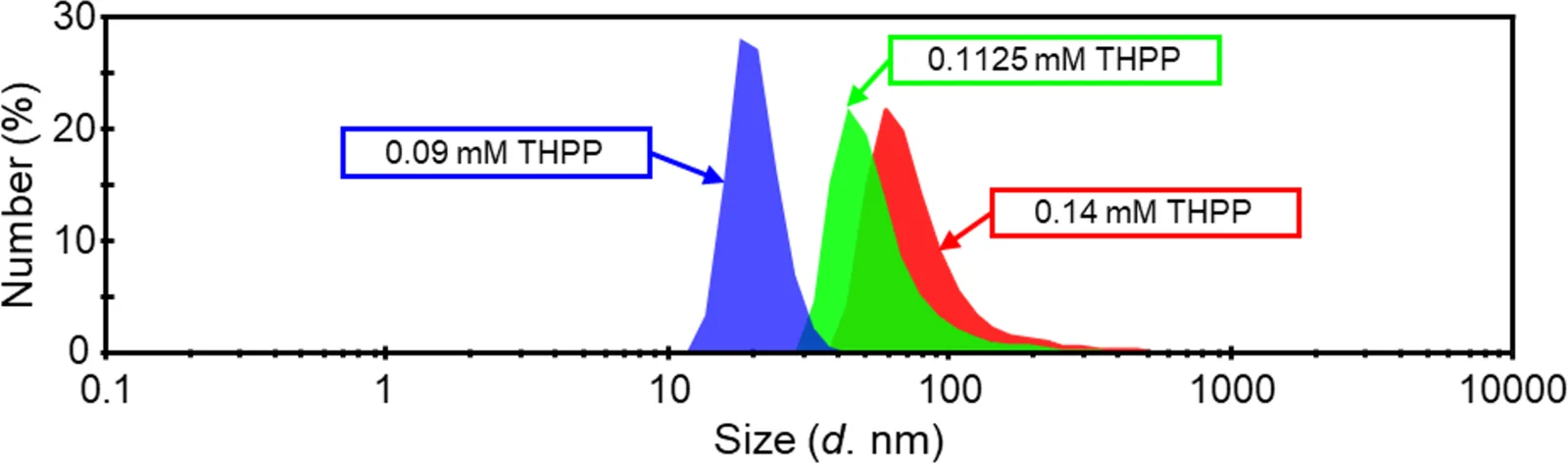
Optimization of nanoparticle formulation parameters showing hydrodynamic diameter distributions from DLS by number for THPP@p(MVCA-BAC) nanoparticles prepared with different initial THPP concentrations of 0.09, 0.1125, and 0.14 mM. Three measurements were taken for all samples.

THPP loading was evaluated at three concentrations of 0.09, 0.1125, and 0.135 mM. At higher THPP concentrations, the sample became insoluble in 15 μL of DMSO containing BAC, while at lower concentrations, the drug loading capacity (LC%) was less than 1%. After self-assembly, free-radical polymerization was performed at 70 °C using ammonium persulfate (2 wt %, relative to total monomer mass). The product was purified by dialysis (90 min with water replaced with distilled water every 30 min, threshold value 3 kDa). Under these conditions, the encapsulation efficiency (EE%) remained modest (20–25%), and LC% did not exceed 1.2% (Table 1). Although low compared to the literature [23], this LC% value might be beneficial, as it allows THPP to remain in a monomeric state within the nanocarrier core, preventing aggregation and thereby positively impacting its photodynamic properties. Furthermore, the particle size increased with THPP concentration, with hydrodynamic diameters ranging from 20 to 60 nm according to dynamic light scattering (DLS) data (number distribution) (Table 1, Figure 2).

**Table 1:** Optimization of formulation parameters for THPP@p(MVCA-BAC) nanoparticles including the effect of polymerization temperature and THPP concentration on particle size (*D*, number distribution), polydispersity index (PDI), encapsulation efficiency (EE%), and drug loading capacity (LC%).^a^

*C*(MVCA-OAcr), mM	*C*(BAC), mM	*V*(DMSO), mL	PDI	*T*_p_, °C	*D*, nm	*C*(THPP)^b^, mM	EE%	LC%

0.5	2.5	15	0.31	70	20.1 ± 3.1	0.09	24.30	0.87
0.5	2.5	15	0.256	70	43.8 ± 4.6	0.1125	23.85	1.07
0.5	2.5	15	0.275	70	58.8 ± 0.7	0.14	20.75	1.12
0.5	2.5	15	0.395	25	38.3 ± 1.7	0.14	40.35	2.15

^a^Three measurements were taken for all samples. ^b^Initial THPP concentrations.

**Figure 3:**
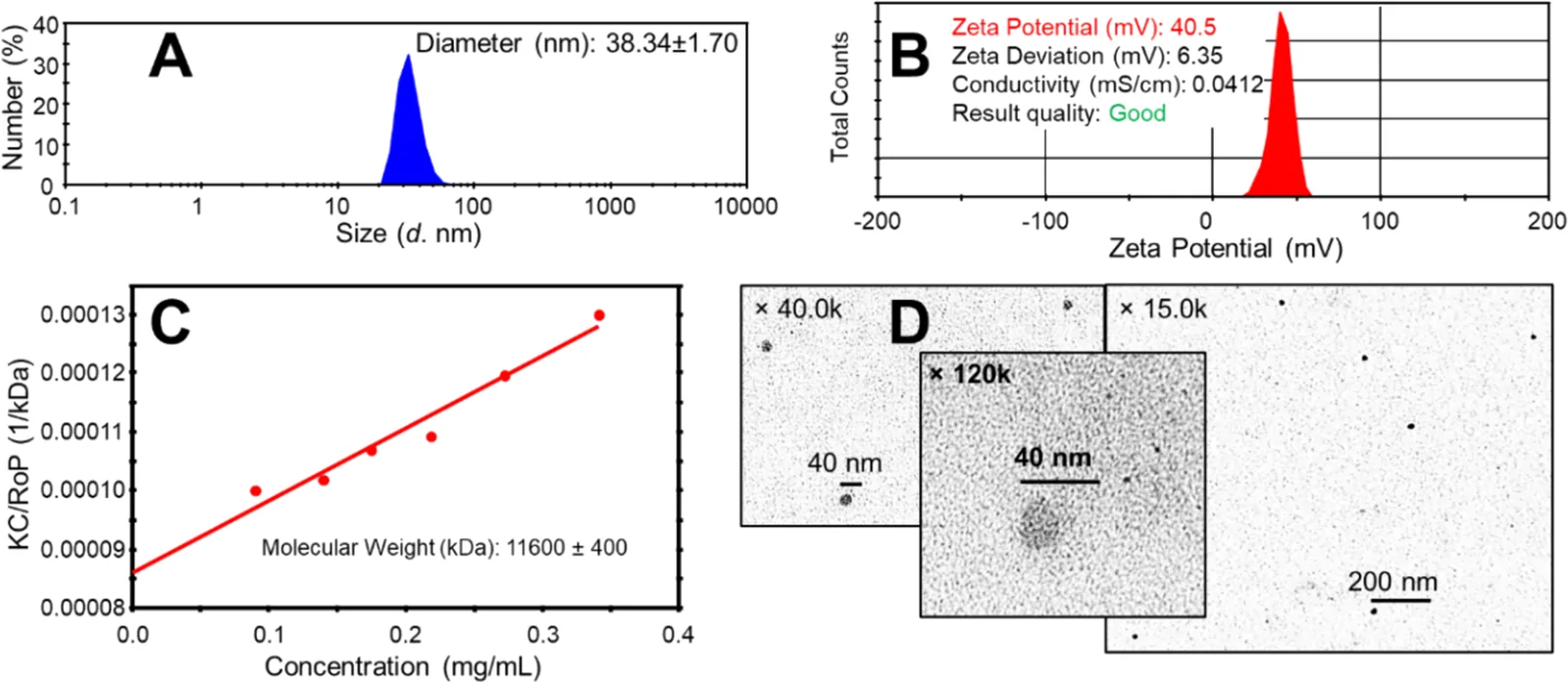
Data for THPP@p(MVCA-BAC): (A) Hydrodynamic size distribution determined by DLS; three measurements were taken. (B) Zeta potential distribution in aqueous media. (C) Debye plot for molecular weight estimation via SLS. (D) TEM images. All measurements were performed in 10 mM phosphate buffer (PB, pH 7.4) at 25 °C. *C*(THPP@p(MVCA-BAC)) = 2 mg/mL.

The reaction temperature had a critical impact on both size and payload. Polymerization at room temperature (25 °C) yielded nanoparticles with a hydrodynamic diameter of 38.3 ± 1.7 nm (Figure 3A). This is significantly smaller than particles made at 70 °C (58.8 ± 0.7 nm, Table 1).

The composite demonstrates good colloidal stability with a zeta potential of +40.5 mV, indicating surface localization of viologen units and preferential interior placement of THPP within the nanoparticles (Figure 3B). This high positive zeta potential allows the nanocarrier to interact electrostatically with negatively charged tumour endothelial and cancer cell membranes, facilitating adsorptive endocytosis and overcoming passive EPR effect limitations. The molecular weight was estimated from static light scattering (SLS) data using a Debye plot (*KC*/*R*₀*P* vs concentration). Extrapolation to zero concentration yields an intercept of 1/*M*, corresponding to a molecular weight of 11600 ± 400 kDa (Figure 3C). More importantly, the milder conditions dramatically improved photosensitizer retention. EE% rose from 20.75% to 40.35%. LC% increased from 1.12% to 2.15% (Table 1). At lower temperatures, the polymerization and self-assembly processes occur at a more controlled, slower rate with reduced thermal motion, allowing the polymer chains to form a more thermodynamically stable and densely packed core around the hydrophobic THPP. Furthermore, elevated temperatures cause accelerated chain movement and chaotic aggregation into larger, looser structures; therefore, the low-temperature protocol yields smaller, more compact particles despite their higher THPP loading capacity. Transmission electron microscopy (TEM) confirmed the formation of monodisperse, spherical nanoparticles. The dry core diameter was ≈25 nm (Figure 3D). This aligns well with the DLS-derived hydrodynamic diameter of about 38 nm. The ≈13 nm difference reflects a hydrated corona rich in viologen groups. This shell extends into the aqueous medium, enhancing colloidal stability. The storage stability of the THPP@p(MVCA-BAC) formulation was assessed, revealing satisfactory long-term colloidal stability. When refrigerated at 4 °C after dialysis purification, the colloid maintained a constant hydrodynamic diameter for at least two months, with no aggregation or precipitation.

An empty p(MVCA-BAC) nanocarrier without THPP (Supporting Information File 1, Scheme S1) was also prepared under the same conditions to serve as a control for comparative analysis of physicochemical properties and biological activity. Its hydrodynamic diameter is about 47.0 ± 9.6 nm (Supporting Information File 1, Figure S1A). The zeta potential is less than that of THPP@p(MVCA-BAC), measuring approximately +17.6 mV (Supporting Information File 1, Figure S1B), and the molecular weight is 10400 ± 570 kDa (Supporting Information File 1, Figure S1C). These findings suggest that the encapsulation of THPP promotes core compaction and structuring, whereas looser particles are formed in its absence.

**Figure 4:**
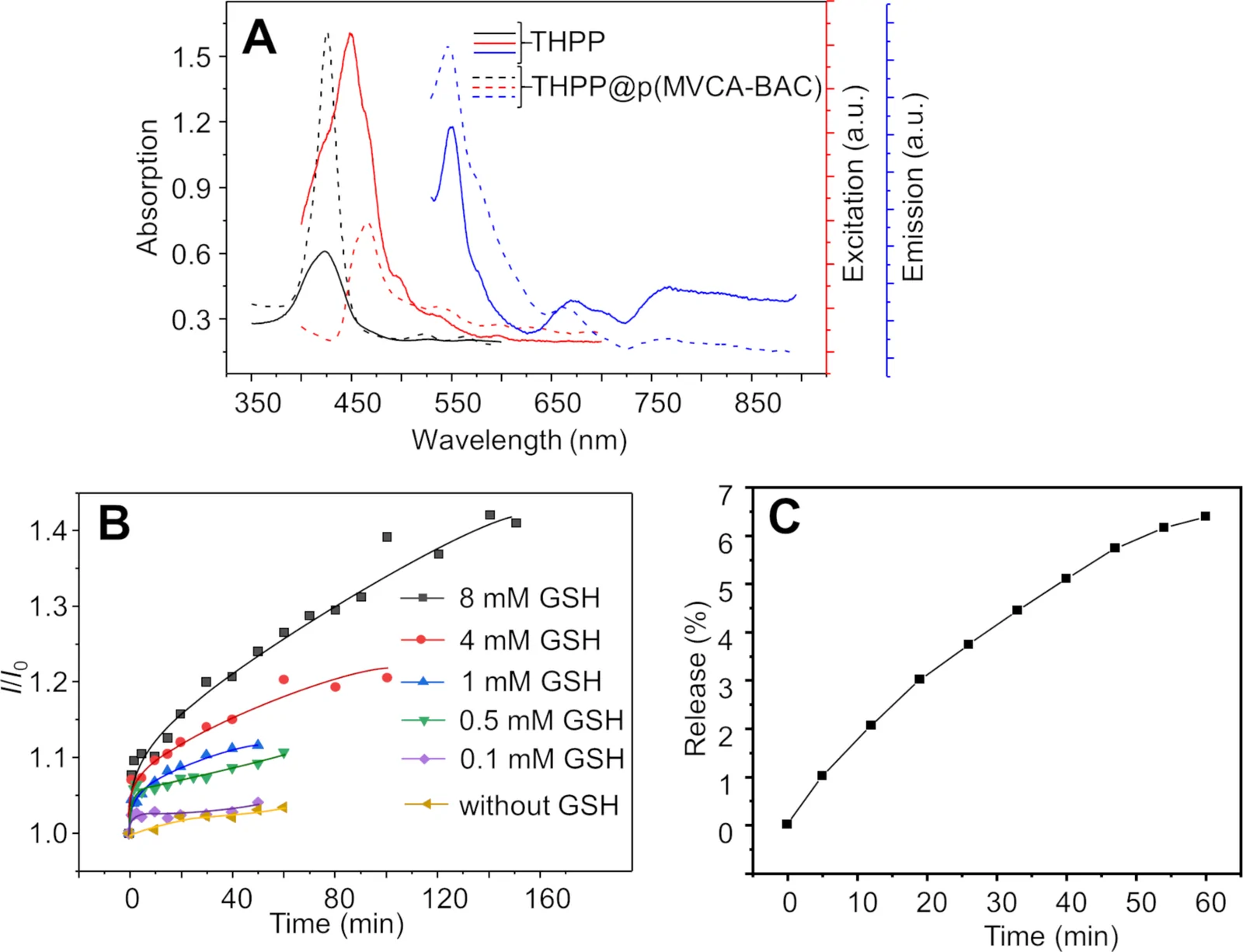
(A) Optical characterization of THPP and THPP@p(MVCA-BAC) in PB pH 7.4: UV–vis absorption (black), fluorescence excitation (red), and emission (blue) spectra *C*(THPP) = 3 μM at 37 °C. (B, C) Redox-triggered release of THPP from THPP@p(MVCA-BAC) in the presence of GSH: (B) Fluorescence data *C*(THPP) = 3 μM, *C*(GSH) = 0–8 mM, pH 6.3, 37 °C. (C) Dialysis data, *C*(THPP) = 3 μM, *C*(GSH) = 8 mM, pH 6.3, 37 °C.

Free THPP exhibits a sharp Soret band at 426 nm in DMSO, reflecting its monomeric state in this organic solvent (Supporting Information File 1, Figure S2). However, in phosphate buffer (PB, pH 7.4), it rapidly aggregates, leading to a broadened and significantly weakened absorption profile (Supporting Information File 1, Figure S2). In contrast, THPP@p(MVCA-BAC) retains a sharp, intense Soret peak at 426 nm, which is nearly identical to its spectrum in DMSO. This confirms efficient solubilisation and disaggregation of the drug within the nanoparticles, whereas pure porphyrin suffers from a dramatic drop in absorbance in an aqueous medium due to aggregation (Figure 4A).

The fluorescence behaviour further supports this conclusion. The excitation spectrum of free THPP exhibits a relatively high intensity, likely due to light scattering or residual absorption by aggregates. However, its emission is severely quenched, reflecting efficient non-radiative decay in the aggregated state. In contrast, the encapsulated THPP exhibits enhanced emission intensity (Figure 4A), suggesting suppressed aggregation-induced quenching and preservation of the photosensitizer’s photoactive state.

### Redox-triggered THPP release

Cancer cells maintain an intracellular GSH concentration of 2–10 mM, far above the 2–20 μM levels in healthy tissues. This reductive environment contributes to drug resistance but can also be exploited for tumour-selective drug release [37]. Our nanocarrier leverages this feature. It incorporates disulfide crosslinks derived from BAC. These bonds remain stable in circulation but undergo rapid cleavage in the presence of elevated GSH. This triggers the disassembly of the nanoparticle core and the release of encapsulated THPP. DLS analysis revealed GSH-triggered disassembly of empty p(MVCA-BAC). After 1 h with 8 mM GSH, the hydrodynamic diameter increased to 122 ± 14 nm (number distribution), indicating a structural transition (Supporting Information File 1, Figure S1A). This demonstrates that reductive disulfide cleavage degrades the dense nanoparticle core into looser ensembles. This matrix deconstruction should enhance carrier permeability, while the resulting amphiphilic polymer fragments can still form supramolecular aggregates to stabilize released THPP and prevent its precipitation in water.

Fluorescence-based THPP release studies in PB (pH 6.3) confirmed the nanocarrier’s redox-responsive behaviour. Without GSH, the THPP fluorescence intensity in the THPP@p(MVCA-BAC) colloid remained stable. However, upon addition of GSH, the emission intensity increased in a strictly concentration-dependent manner across a range of 0.1, 0.5, 1, 4, and 8 mM (Figure 4B). Specifically, 8 mM GSH triggered a 1.4-fold increase in THPP emission intensity over 2.5 h. This concentration-dependent dequenching suggests that higher GSH levels induce a greater degree of disulfide bond cleavage, which disrupts the polymeric network, makes the nanocarrier structure looser, and provides greater molecular freedom for the encapsulated THPP.

UV–visible spectroscopy (Figure 4C) showed that only about 7% of THPP was released over the course of 1 h during a dialysis experiment. This low release rate is probably due to THPPs poor water solubility and its tendency to remain within the MVCA-based ensemble formed after nanocarrier degradation.

### Efficient singlet oxygen generation by THPP@p(MVCA-BAC) under red-light irradiation

**Figure 5:**
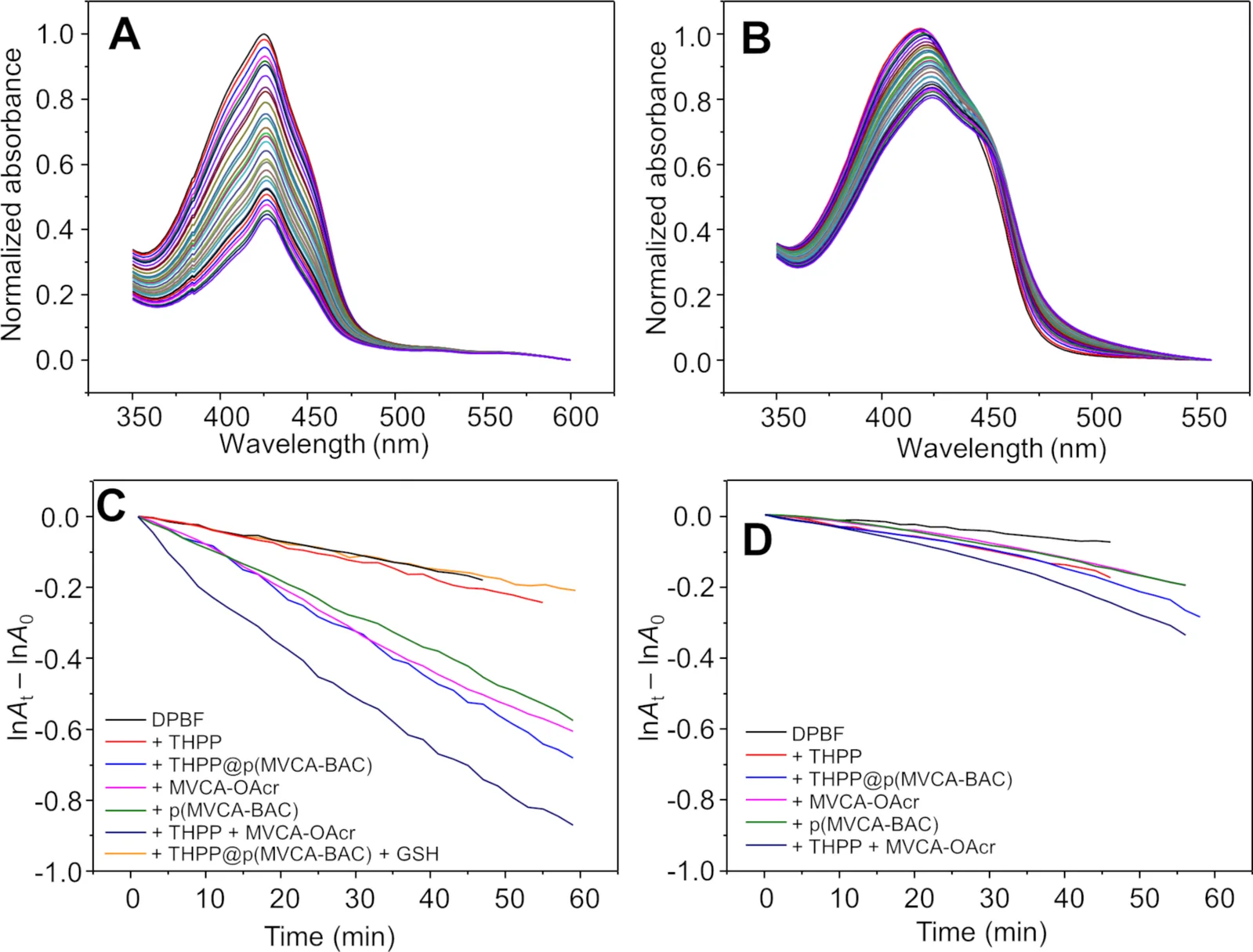
^1^O_2_ generation monitored via DPBF degradation: (A, B) UV–vis spectral changes of DPBF upon 650 nm irradiation (5 mW) in the presence of (A) THPP@p(MVCA-BAC) and (B) free THPP (0.3 μM THPP equivalent) in PB pH 7.4. (C, D) Time-dependent decay of DPBF absorbance at 414 nm fitted to pseudo-first-order kinetics: (C) under irradiation 650 nm, 5 mW and (D) in the dark, *C*(DPBF) = 30 μM, *C*(THPP) = 0.3 μM, PB pH 7.4, 25 °C.

Photodynamic experiments were performed at 650 nm, which closely matches the Q-band maximum of THPP at 654 nm (Figure 4A). This wavelength lies within the phototherapeutic window (600–900 nm), enabling deep tissue penetration (>5 mm) while minimizing damage to healthy cells [38]. A red laser diode (650 nm, 5 mW) was used for all studies. Singlet oxygen (^1^O_2_) generation was monitored using 1,3-diphenylisobenzofuran (DPBF) as a chemical trap. Despite the low aqueous solubility of DPBF, a stable dispersion was achieved by rapidly adding a concentrated solution of DPBF in DMSO to diluted analyte solutions, enabling kinetic measurements in PB (pH 7.4). Under irradiation, THPP@p(MVCA-BAC) caused a rapid decrease in DPBF absorbance at 414 nm, indicative of ^1^O_2_-mediated oxidation (Figure 5A). The reaction followed pseudo-first-order kinetics with a rate constant of *k* = 0.0121 ± 0.0001 min^−1^ (Table 2). This is over 3.5-fold higher than the background degradation observed for DPBF alone under identical irradiation (*k* = 0.0035 min^−1^). The slow decay in the control likely reflects DPBF the limited solubility and subsequent precipitation of DPFB. Notably, free THPP in the presence of DPBF under the same conditions showed no significant photosensitizing activity, and the resulting kinetic profile was nearly identical to that of DPBF alone (Figure 5B). This confirms that free THPP undergoes severe aggregation in the purely aqueous medium, which suppresses its photodynamic capacity. In both cases, the spectra revealed a bathochromic shift accompanied by peak broadening rather than a substantial decrease in absorbance intensity. This spectral behaviour is presumably driven by the self-aggregation and gradual precipitation of DPBF in the aqueous medium, which occurs due to its poor water solubility and is accelerated under irradiation.

**Table 2:** Pseudo-first-order rate constants (*k*, min^−1^) for DPBF degradation under 650 nm irradiation (5 mW) in PB pH 7.4, *C*(DPBF) = 30 µM, *C*(THPP) = 0.3 μM, 25 °C.

Sample	*k* (min^−1^)

DPBF only	0.00347 ± 0.00004
THPP	0.00460 ± 0.00005
p(MVCA-BAC) (no THPP)	0.00981 ± 0.00005
MVCA-OAcr	0.01100 ± 0.00001
THPP@p(MVCA-BAC)	0.01206 ± 0.00009
THPP + MVCA-OAcr (physical mix)	0.01452 ± 0.00003
THPP@p(MVCA-BAC) + GSH	0.00366 ± 0.00004

Interestingly, the kinetic profiles of free MVCA-OAcr (*k* = 0.0110 min^−1^), empty p(MVCA-BAC) (*k* = 0.0098 min^−1^), and encapsulated THPP@p(MVCA-BAC) (*k* = 0.0121 min^−1^) are relatively similar. This indicates that DPBF potentially interacts and coaggregates with the surface viologen units present across all these systems, leading to spectral artefacts that mask the true singlet oxygen generation in cell-free aqueous media. Nevertheless, the physical mixture of free THPP and MVCA-OAcr exhibited the highest DPBF decay rate (*k* = 0.0145 min^−1^) (Table 2, Figure 5C,D). This suggests that MVCA-OAcr improves the THPP solubility through assembly, thereby enhancing its photodynamic activity. To evaluate the effect of GSH on singlet oxygen generation, THPP@p(MVCA-BAC) was pre-incubated with GSH (8 mM) for 1 h prior to the addition of DPBF, allowing GSH-triggered degradation and THPP release. This led to a sharp decrease in the rate constant to *k* = 0.00366 ± 0.00004 min^−1^ (Table 2, Figure 5A), which is nearly identical to the background DPBF degradation. This drop occurs because glutathione acts as a powerful antioxidant in solution, directly scavenging the generated singlet oxygen and competing with the DPBF trap. However, the cellular environment is more complex than a simple aqueous solution, so photodynamic activity might still occur despite intracellular antioxidants.

### Biological investigations

#### Selective photodynamic activity and real-time monitoring of cellular response

PDT is primarily suited for accessible malignancies, including superficial lesions and tumors reachable via endoscopy. Given that solid tumors are characterized by elevated intracellular GSH levels, the in vitro cytotoxicity of p(MVCA-BAC) and THPP@p(MVCA-BAC) was evaluated across a broad panel of cancer models, including breast (MBA-MD-231), cervical (M-HeLa), colorectal (Caco-2), prostate (PC-3), melanoma (A375), and duodenal (HuTu-80) cells. Non-malignant WI-38 and HBL-100 lines were used as healthy controls (Table 3).

**Table 3:** Dark cytotoxicity (IC_50_, µM) of p(MVCA-BAC) and THPP@p(MVCA-BAC) against malignant and non-malignant cell lines.^a^

	p(MVCA-BAC) (µM)	THPP@p(MVCA-BAC) (µM by polymer)	THPP@p(MVCA-BAC) (µM by THPP)

HBL-100	>50	>50	>4.4
Caco-2	>50	>50	>4.4
A375	>50	>50	>4.4
M-HeLa	>50	>50	>4.4
PC-3	>50	37.1 ± 0.2	3.3 ± 0.02
BT-20	>50	41.6 ± 0.1	3.7 ± 0.01
MBA-MD-231	50.5 ± 2.0	28.3 ± 0.1	2.5 ± 0.01
HuTu-80	27.0 ± 1.8	32.6 ± 0.6	2.9 ± 0.05
WI-38	32.7 ± 0.2	27.0 ± 0.2	2.4 ± 0.02

^a^Average of three values measured; ± standard deviation (SD).

Table 3 shows that both p(MVCA-BAC) and THPP@p(MVCA-BAC) have low toxicity (IC_50_ > 50 μM by polymer) in HBL-100, Caco-2, A375, and M-HeLa cell lines. THPP encapsulation slightly decreased viability (IC_50_ 28.3–41.6 μM by polymer) in PC-3, BT-20, and MBA-MD-231 lines. HuTu-80 and WI-38 models were more sensitive to the polymer matrix. These results confirm a good dark safety profile, crucial for minimizing PDT side effects. Notably, the precursor MVCA-OH monomer (Supporting Information File 1, Scheme S1) exhibited a similarly low cytotoxicity (Supporting Information File 1, Table S2), falling within the same high micromolar range as the p(MVCA-BAC) nanocarrier. Based on these profiles, the formulation is primarily intended for direct intratumoral injection, though intravenous administration remains possible.

To evaluate safety upon contact with blood, hemolytic activity was investigated. Both p(MVCA-BAC) and THPP@p(MVCA-BAC) exhibited minimal hemolytic activity, with hemolysis remaining below 10% even at the highest tested concentrations (HC_50_ > 0.05 mM) (Supporting Information File 1, Table S3). This is acceptable blood compatibility, despite the positive surface charge.

**Figure 6:**
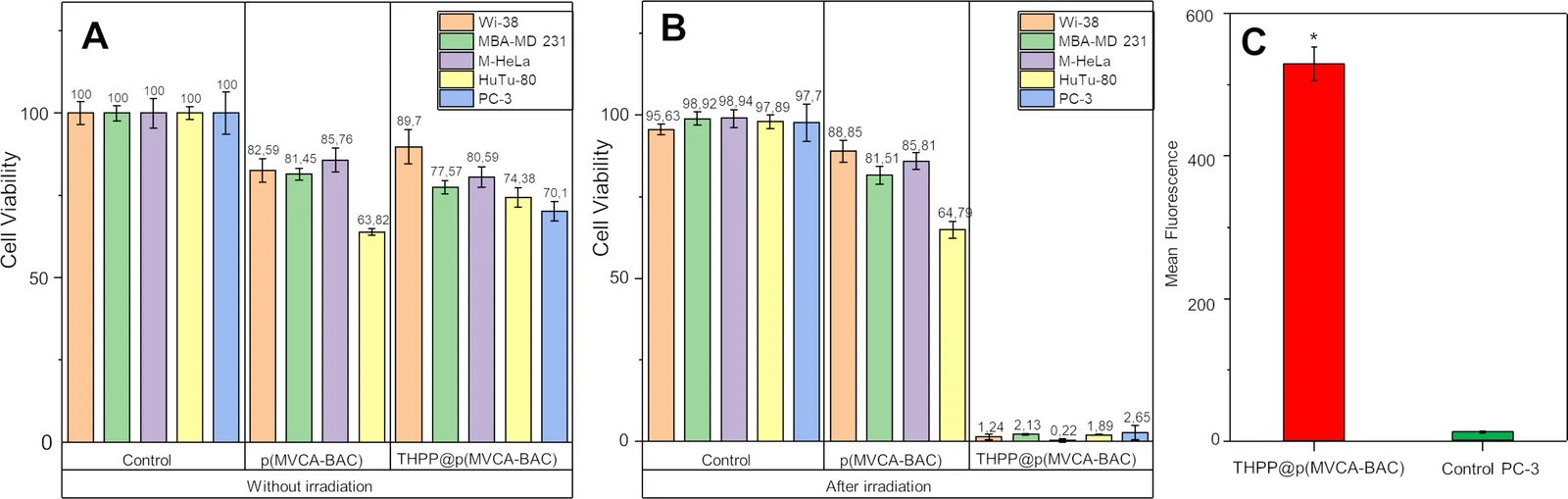
(A, B) Cell viability after treatment with p(MVCA-BAC) and THPP@p(MVCA-BAC): (A) in the dark and (B) after 650 nm irradiation (60 mW, 3 min), *C*(THPP) = 2 μM. (С) Cellular uptake study of THPP@p(MVCA-BAC) by flow cytometry method, *C*(THPP) = 2.7 μM, Control - intact PC-3 cells, *values are given at *P* < 0.01, data are mean ± SD (*n* = 3).

For photophysical studies, a lower irradiance (5 mW) was used to determine ^1^O_2_ via DPBF, ensuring controlled reaction kinetics. For biological phototoxicity, a clinically relevant irradiance of 60 mW was applied for 3 min for efficient tumour cell ablation. At 2.0 µM, THPP@p(MVCA-BAC) exhibited mild, concentration-dependent dark toxicity (65–85% cell viability; Figure 6A). However, irradiation with 650 nm light for 3 min resulted in severe photodamage, reducing cell viability to 0.22–2.65% (Figure 6B). This significant difference between dark safety and light-induced cytotoxicity confirms the photodynamic efficiency and wide therapeutic window of THPP@p(MVCA-BAC) for tumour ablation.

Encapsulation of THPP within p(MVCA-BAC) dramatically enhances its cellular uptake. Free THPP, due to its poor aqueous solubility, shows negligible internalization under physiological conditions. In contrast, treatment with THPP@p(MVCA-BAC) at a THPP concentration of 2.7 μM resulted in a 35-fold increase in intracellular fluorescence intensity, as quantified by flow cytometry (Figure 6С). This remarkable improvement stems from the synergistic design of the nanocarrier: The hydrophobic core effectively solubilizes THPP in its monomeric, photoactive form, while the viologen-rich surface promotes interactions with the negatively charged cell membrane and facilitates endocytic uptake. The magnitude of this enhancement exceeds typical values reported for porphyrin-based nanodelivery systems [39–40], highlighting the ability of p(MVCA-BAC) to overcome the fundamental bioavailability barrier that limits the clinical utility of hydrophobic photosensitizers. Together, these results demonstrate that THPP@p(MVCA-BAC) acts as a tumour-selective PDT agent exhibiting low toxicity in the dark but potently cytotoxic upon light activation.

#### Real-time monitoring of photodynamic response

**Figure 7:**
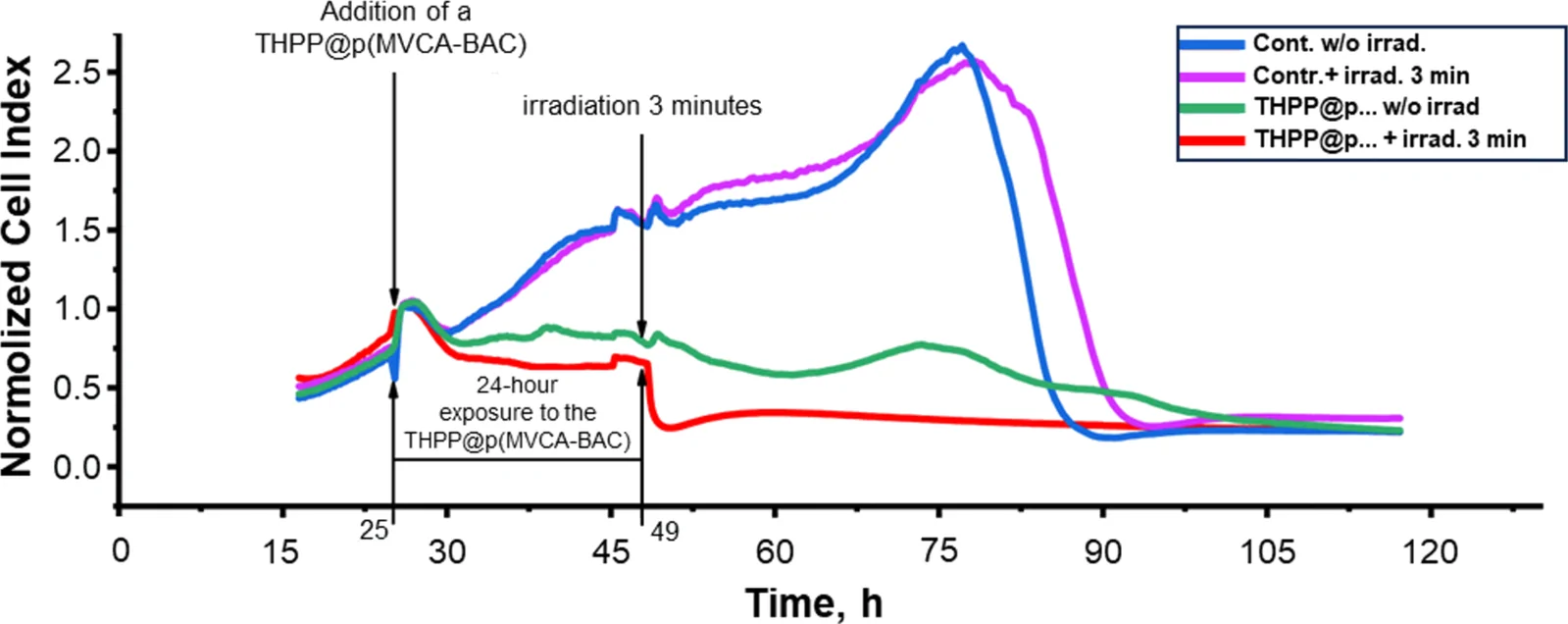
Real-time monitoring of the PC-3 cell response to THPP@p(MVCA-BAC) using impedance-based proliferation assay, cells were treated at 26 h with THPP@p(MVCA-BAC) (2.7 μM THPP) and irradiated at 49 h with 650 nm light (60 mW, 3 min); traces: (blue) untreated control; (violet) light only; (green) THPP@p(MVCA-BAC) in the dark; (red) THPP@p(MVCA-BAC) + light, data are mean ± SD (*n* = 4).

Real-time cell proliferation kinetics were monitored using impedance-based sensing (xCELLigence) after treatment of PC-3 cells with THPP@p(MVCA-BAC) (2.7 μM THPP). In untreated controls (blue trace, Figure 7), cells exhibited exponential growth until nutrient exhaustion at ≈78 h, followed by gradual decline.

Irradiation alone (650 nm, 60 mW, 3 min at 49 h) had no detectable effect on proliferation (violet trace), confirming the absence of phototoxicity in the absence of the photosensitizer. Non-irradiated cells treated with THPP@p(MVCA-BAC) (green trace) exhibited a stabilization of the cell index within 24 h post-treatment, with minimal cell death (<10%). This plateau likely reflects a transient cytostatic phase rather than acute toxicity, which could be attributed to a temporary cell cycle arrest induced by the metabolic stress of active nanoparticle endocytosis [41]. In sharp contrast, light activation (650 nm, 60 mW, 3 min at 49 h) triggered immediate and massive cell death: ≥95% of cells were eliminated within 2 h (red trace). This rapid photodynamic ablation matches the kinetics observed with other porphyrin-based PDT agents [42–43] and underscores the spatiotemporal precision of our system. Together, these results reveal a dual functionality of THPP@p(MVCA-BAC), that is, (1) a dark cytostatic effect that halts cell division without overt toxicity, and (2) a light-triggered cytotoxic switch enabling on-demand tumor cell eradication.

#### Flow cytometry analysis of apoptosis induction

**Figure 8:**
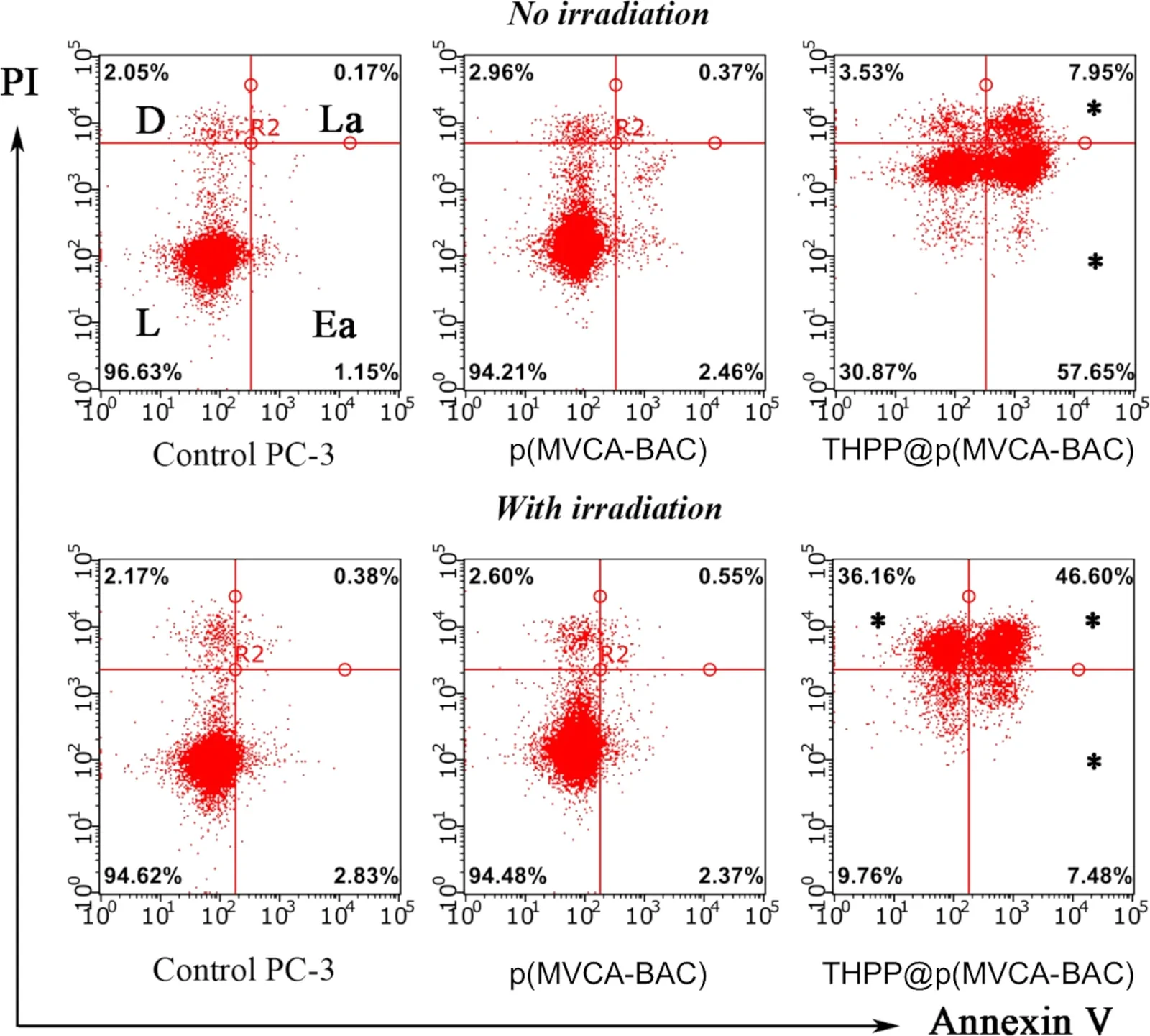
Induction of apoptosis in PC-3 cells incubated with p(MVCA-BAC) and THPP@p(MVCA-BAC), *C*(THPP) = 2 µM, for 24 h. Apoptotic effects were measured by flow cytometry using the annexin V – Elab Fluor 647/PI staining protocol. L – live cells; D – dead cells; Ea – cells at the early stage of apoptosis; La – cells at the late stage of apoptosis. Values are presented as mean ± standard deviation. Experiments were repeated three times. *p < 0.01 compared to control.

The apoptosis-inducing capacity of empty p(MVCA-BAC) and THPP@p(MVCA-BAC) was evaluated on PC-3 cells using flow cytometry (Figure 8). It was established that the empty p(MVCA-BAC) does not enhance apoptosis induction: The number of cells in the early and late stages of apoptosis did not significantly differ from the control, both during incubation in the dark and after irradiation.

Conversely, cultivating cells with THPP@p(MVCA-BAC) in the dark led to the active induction of apoptosis, predominantly dominated by early apoptotic events (57.65%), while the fraction of dead cells remained negligible (3.53%) and comparable to the control. Under irradiation, a completely different pattern was observed: The majority of the cells (46.60%) transitioned into the late stage of apoptosis, and a significant fraction of dead cells (36.16%) also emerged. These results indicate that the cytotoxic effect of the THPP@p(MVCA-BAC) system in the dark mode is primarily driven by its early apoptosis-inducing activity. The addition of irradiation engages an additional mechanism leading to cancer cell death, which is associated with the photodynamic generation of ROS by the encapsulated THPP.

#### Intracellular uptake and subcellular localization

**Figure 9:**
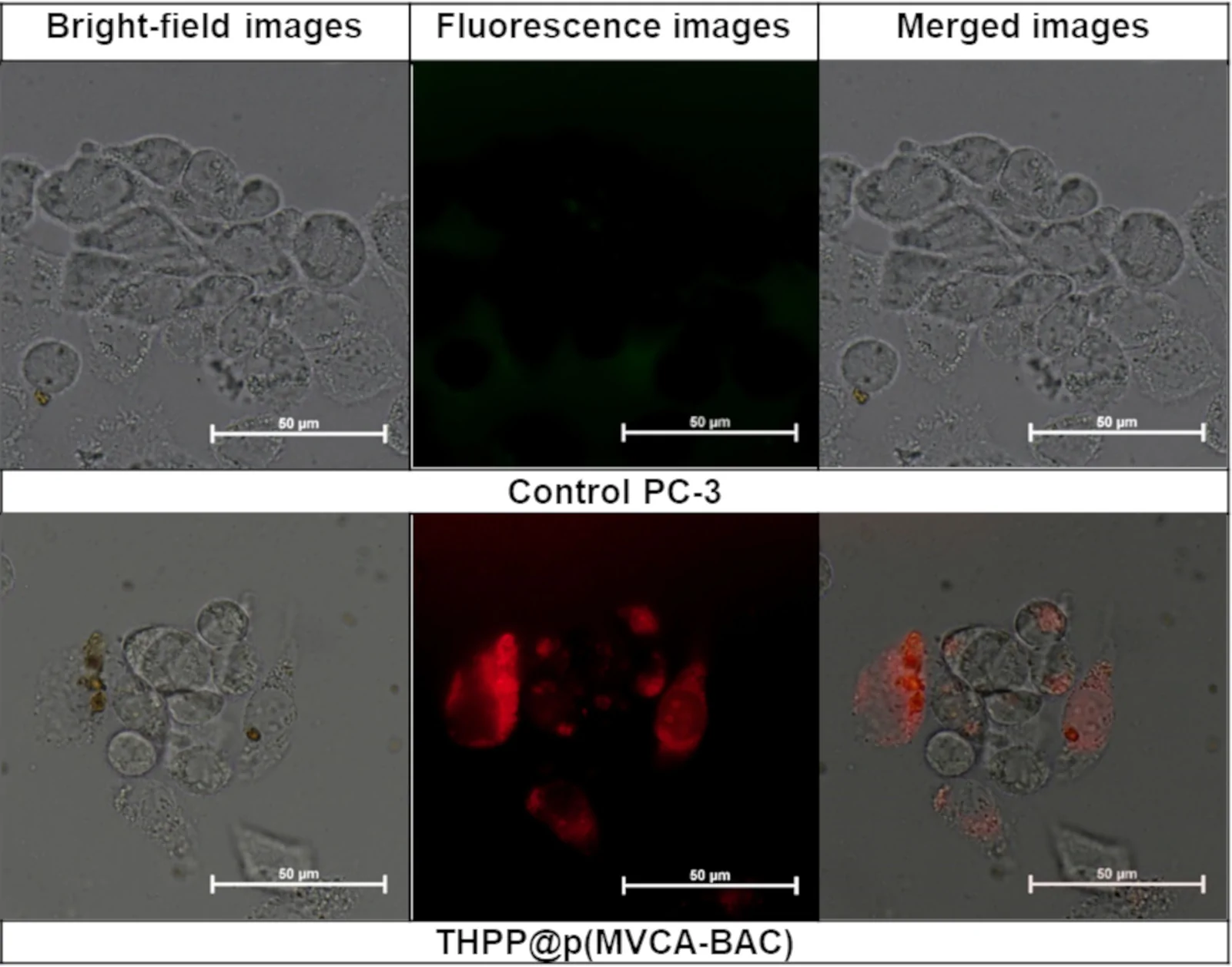
Cellular uptake and localization of THPP@p(MVCA-BAC) in PC-3 cells, bright-field, fluorescence, and merged images after 24 h incubation with THPP@p(MVCA-BAC) (2.7 μM THPP) or untreated control, scale bar: 50 μm.

Cellular uptake and subcellular localization of THPP@p(MVCA-BAC) were assessed by fluorescence microscopy combined with bright-field imaging (Figure 9). The overlay of fluorescence and transmitted-light images ensures reliable interpretation and minimizes false-positive signals. Bright-field images confirmed that PC-3 cells remained morphologically intact after 24 h incubation with THPP@p(MVCA-BAC) (2.7 μM THPP), with no signs of membrane blebbing, rounding, or detachment, indicative of preserved viability in the dark. In contrast, strong red fluorescence, characteristic of THPP, was observed throughout the cytoplasm, confirming efficient internalization of the nanocarrier. No signal was detected in untreated control cells.

These results corroborate flow cytometry data and demonstrate that THPP@p(MVCA-BAC) is readily taken up by prostate cancer cells and accumulates in the cytoplasm, positioning the photosensitizer in proximity to key organelles for effective photodynamic action upon irradiation. Together, these results establish THPP@p(MVCA-BAC) as a tumor-selective, activatable PDT platform: It remains benign until illuminated, at which point it unleashes potent, localized cytotoxicity, fulfilling key criteria for clinical PDT agents operating within the 650–900 nm therapeutic window.

#### Light-triggered intracellular ROS burst mediates photocytotoxicity

**Figure 10:**
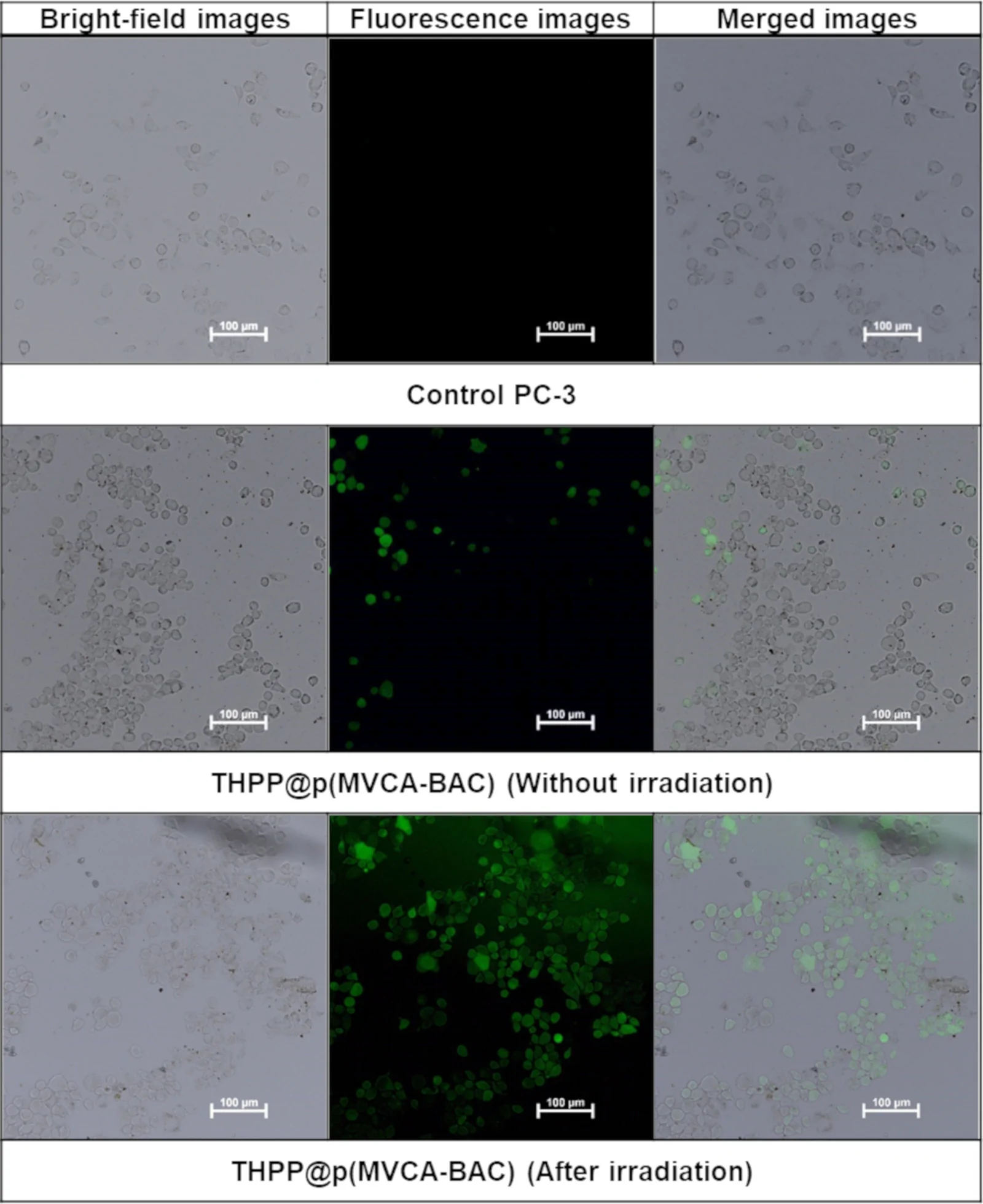
Light-triggered intracellular ROS generation in PC-3 cells, H2DCFDA fluorescence (green) after treatment with THPP@p(MVCA-BAC) (2.7 µM THPP) with or without 650 nm irradiation (60 mW, 3 min), scale bar: 100 μm.

To directly verify the mechanism of cell death, intracellular ROS generation was monitored in PC-3 cells using H2DCFDA, a cell-permeable probe that fluoresces upon oxidation by reactive oxygen species. As expected, untreated control cells showed negligible DCF fluorescence, confirming low basal ROS levels (Figure 10).

Cells incubated with THPP@p(MVCA-BAC) (2.7 μM THPP) in the dark exhibited only a modest increase in fluorescence, consistent with mild oxidative stress, possibly arising from redox-active viologen groups on the nanoparticle surface. In stark contrast, immediate and intense green fluorescence was observed across the entire cell population within minutes after 650 nm irradiation (60 mW, 3 min), indicating a massive, light-triggered ROS burst. This ROS surge directly correlates with the rapid cell death observed in real-time impedance assays (Figure 7) and confirms that photocytotoxicity is mediated by oxidative damage. Critically, the response is light-dependent and photosensitizer-specific: No significant signal was detected in irradiated controls without the nanocarrier. This on-demand activation underscores the precision of THPP@p(MVCA-BAC) as a PDT agent.

## Conclusion

We developed a redox-responsive THPP@p(MVCA-BAC) nanoplatform assembled via the copolymerization of a viologen-cavitand and a disulfide crosslinker. This system stabilizes hydrophobic THPP in its monomeric, non-aggregated state, maintains colloidal stability for at least three months, and enables GSH-triggered payload release. In vitro screening across six malignant and two non-malignant cell lines, combined with hemocompatibility assays, confirmed the low toxicity profile of the formulation. The nanocarrier exhibits a dual action, namely, a reversible cytostatic effect driven by early apoptosis in the dark and efficient cell death under 650 nm irradiation. Flow cytometry validated that laser activation drives a transition from early dark cytostasis to late apoptosis and direct cell destruction via an intracellular ROS burst. This combination of supramolecular templating and redox responsiveness provides a reliable approach for the delivery of photodynamic agents.

## Experimental

### Instrumentation

TEM images were acquired on a Zeiss Libra 120 EFTEM (Carl Zeiss SMT AG, Oberkochen, Germany) at 100 kV. Samples were prepared by depositing a nanoparticle dispersion onto 300-mesh copper grids coated with a carbon/Formvar support film, followed by air drying. Hydrodynamic diameter and PDI were measured by DLS. Zeta potential was determined by electrophoretic light scattering (ELS). Molecular weight was estimated by SLS using Debye plot analysis. All measurements were performed on a Malvern Zetasizer Nano ZS equipped with ZS Xplorer software. UV–vis absorption spectra were recorded on a PerkinElmer Lambda 25 spectrophotometer. THPP concentrations were determined using the Soret band absorbance at 425 nm and a molar extinction coefficient of ε = 4.45 × 10^5^ M^−1^·cm^−1^. Steady-state fluorescence spectra were obtained on a Hitachi F-7100 spectrofluorometer using 1 cm path-length quartz cuvettes. Excitation spectra were recorded by monitoring emission at 750 nm while scanning excitation from 400 to 700 nm. Emission spectra were collected with excitation at 465 nm and emission scanned from 500 to 900 nm. IR spectra were acquired on a Bruker Tensor 27 spectrometer using KBr pellets (4000–400 cm^−1^). pH values were measured using a Thermo Scientific Orion 2-Star pH meter calibrated with standard buffers. Cyclic voltammetry (CV) was performed using a P-45X potentiostat (Elins, Russia) in a standard three-electrode cell under argon atmosphere. The working electrode was a glassy carbon disk (2 mm diameter), polished before each experiment with 0.05 μm alumina slurry. A platinum wire served as the counter electrode, and potentials were referenced to a saturated calomel electrode (SCE). All measurements were carried out in 0.1 M NaCl aqueous solution at 295 K, without *iR* compensation.

### Materials

MVCA-OAcr, THPP, and BAC were synthesized as described previously in [29,44–45], respectively. Ammonium persulfate (Acros Organics), 1,3-diphenylisobenzofuran (DPBF, BLD Pharm), and glutathione (GSH, Sigma-Aldrich) were used as received. PB (pH 6.3 and 7.4) were prepared according to standard protocols [46]. Deionized water (18.2 MΩ·cm) was obtained from an Adrona Crystal E purification system and used for all aqueous solutions.

### Synthesis of THPP@p(MVCA-BAC)

0.65 mg (2.5 μM) of BAC was dissolved in 15 μL of 9.0 mM solution of THPP in DMSO and then was rapidly injected into 1.0 mL of an aqueous MVCA-OAcr solution (0.50 mM) under vigorous stirring, followed by simultaneous sonication and argon purging for 90 min. Polymerization was initiated by addition of ammonium persulfate (2 wt % relative to total monomer mass) and allowed to proceed at 25 °C for 18 h. The resulting nanoparticles were purified by dialysis against deionized water (1 kDa; 3 × 30 min, with water replacement after each interval) to remove unencapsulated THPP, residual DMSO, and oligomeric byproducts.

LC% and EE% were determined by UV–vis spectroscopy. After dialysis, the concentration of encapsulated THPP was quantified at 425 nm (ε = 4.45 × 10^5^ M^−1^·cm^−1^). LC% and EE% were calculated as:


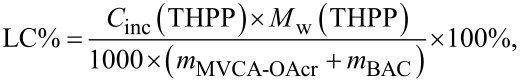


where *C*_inc_(THPP) is the concentration of encapsulated THPP [mM], *M*_w_(THPP) is the molecular weight of THPP [714.8 g/mol], and *m*_MVCA-OAcr_ and *m*_BAC_ are, respectively, the masses of MVCA-OAcr and BAC used in the synthesis.


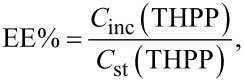


where *C*_st_(THPP) is the concentration of THPP used in the synthesis [mM].

### Synthesis of p(MVCA-BAC)

Empty p(MVCA-BAC) nanoparticles were prepared following the same protocol as for THPP@p(MVCA-BAC), with the exception that BAC in pure DMSO (15 μL) was used in place of the THPP/BAC/DMSO solution (Supporting Information File 1, Scheme S1). All other conditions, monomer concentrations, initiator amount, reaction time, and purification, were identical: hydrodynamic diameter *d* = 47.0 ± 9.6 nm (number distribution), molecular weight *M* = 10400 ± 570 kDa, and zeta potential *ζ* = +17.6 , mV (Supporting Information File 1, Figure S1).

Successful polymerization via the hydroxy group of MVCA-OH (Supporting Information File 1, Scheme S1) was confirmed by the disappearance of the butanolic oxidation peak in cyclic voltammetry (Supporting Information File 1, Figure S3 and Table S1). IR spectroscopy further supported the formation of the polymeric network (Supporting Information File 1, Figure S4).

### Singlet oxygen generation assay

Singlet oxygen generation was evaluated using DPBF as a chemical trap. Aqueous DPBF solutions (30 μM in PB pH 7.4) were prepared by rapid co-injection of a DMSO stock solution into buffer to ensure colloidal stability despite the low aqueous solubility of DPBF. Test samples containing 0.3 μM THPP (free or encapsulated) and/or 2.76 μM MVCA-OAcr containing compounds were added to the DPBF solution. The mixture was irradiated with a 650 nm laser (5 mW), and the decrease in DPBF absorbance at 414 nm was monitored by UV–vis spectroscopy (350–600 nm) over time.

Pseudo-first-order rate constants (*k*) were determined from the initial linear region of ln(*A*_0_/*A*) vs time plots, where *A*_0_ and *A* are the absorbances at 414 nm before and after irradiation, respectively.

For the experiment in the presence of GSH, the THPP@p(MVCA-BAC) nanoparticle solution was first added to solid GSH to achieve a GSH concentration of 8 mM. The mixture was shaken to ensure complete dissolution of GSH and then incubated for 1 h to allow for GSH-triggered nanoparticle disassembly and THPP release. Subsequently, DPBF was introduced, and singlet oxygen generation kinetics were monitored under irradiation.

### Redox-triggered THPP release

The GSH-responsive release of THPP was evaluated in PB pH 6.3 at 37 °C to mimic the reductive tumor microenvironment. A dispersion of THPP@p(MVCA-BAC) (3 μM THPP equivalent) was treated with reduced GSH (8 mM). Fluorescence emission spectra (λ_ex_ = 465 nm, λ_em_ = 600–800 nm) were recorded at defined time intervals over 2.5 h using a 1 cm path-length quartz cuvette.

### Cellular uptake

#### Flow cytometry assay

PC-3 cells were seeded in 24-well plates at a concentration of 1 × 10^5^ cells/well in a final volume of 500 μL. After 24 h of incubation, the wells were supplemented with the porphyrin complex at a concentration of 1:3 and incubated for 24 h in a CO_2_ incubator. The cellular uptake of THPP@p(MVCA-BAC) was analyzed by flow cytometry (Guava easy Cyte 8HT, USA). Flow cytometry was used to obtain statistics on the uptake of the complex by cancer cells. Intact cells were used as a negative control. It was shown that the fluorescence intensity of cells treated with THPP@p(MVCA-BAC) increased compared to the control.

### Real-time cell proliferation monitoring

The cell culture was treated with THPP@p(MVCA-BAC) (ratio 1:3) added to the growth medium. Proliferation kinetics were monitored in real time using the XCELLigence S16 RTCA real-time analysis system (Acea Biosciences, USA) for 115 h.

### Apoptosis induction assay

The apoptosis-inducing capacity of the p(MVCA-BAC) and THPP@p(MVCA-BAC) systems was assessed in PC-3 human prostate adenocarcinoma cells. Cells were incubated with the compounds (*C*(THPP) = 2 µM) for 24 h and then analysed by flow cytometry with double staining with annexin V–Elab Fluor 647 and propidium iodide (PI). Annexin V specifically binds to phosphatidylserine exposed on the outer side of the plasma membrane during the early stages of apoptosis, whereas PI penetrates only cells with impaired membrane integrity, allowing differentiation between late apoptotic cells and necrotic cells.

### Microscopy assay

#### Microscopic analysis

PC-3 cells at a concentration of 1 × 10^6^ cells/well in a volume of 2 mL were seeded in 6-well plates with coverslips at the bottom of each well. After 24 h of incubation, the studied complex was added to the wells at a concentration of 1:3 and cultured for 24 h in a CO_2_ incubator. Photography was performed using a fluorescence module and in transmitted light on a Nikon Eclipse Ci-S microscope (Nikon, Japan) at 1000× magnification.

Penetration of the porphyrin system into PC-3 cells was studied using fluorescence microscopy and bright-field microscopy, since the combination of fluorescence images with transmitted light photographs increases the reliability of data interpretation and excludes false positive results. Bright-field microscopy allows one to assess the general condition of the cells (normal shape, absence of damage, and signs of apoptosis or necrosis). Fluorescence microscopy showed strong red fluorescence of the porphyrin, indicating the presence of the system in significant amounts in PC-3 cells. Transmitted light photographs demonstrated that THPP@p(MVCA-BAC) porphyrin penetration at the tested concentration did not cause serious damage to PC-3 cells. Fluorescence and superimposed photographs show localization of nanoparticles in the cytoplasm of prostate cancer cells.

#### Induction of intracellular ROS production

PC-3 cells were seeded at 1 × 10^5^ cells/well in a final volume of 2 mL in 6-well plates with coverslips at the bottom of each well. After 24 h of incubation, THPP@p(MVCA-BAC) was added to the wells at a concentration of 1:3 and cultured for 24 h in a CO_2_ incubator. Induction of intracellular ROS production was studied using 2′,7′-dichlorofluorescein diacetate (DCFH-DA). 2',7'-dichlorodihydrofluorescein diacetate (H2DCFDA) is a chemically reduced form of fluorescein used as an indicator of reactive oxygen species (ROS) in cells. After cleavage of acetate groups by intracellular esterases and oxidation, non-fluorescent H2DCFDA is converted to highly fluorescent 2',7'-dichlorofluorescein (DCFH-DA). For ROS detection, PC-3 cells were harvested at 2000 rpm for 5 min, then washed twice with ice-cold PBS, resuspended in 0.5 mL of FBS-free growth medium containing 5 μM DCFH-DA, and incubated at 37 °C for 1 h. After washing the cells three times with PBS, ROS production in the cells was immediately monitored using a fluorescence module and in transmitted light on a Nikon Eclipse Ci-S microscope (Nikon, Japan) at 200× magnification.

## Supporting Information

Synthesis and characteristics of p(MVCA-BAC); optical spectra of free THPP in DMSO and PB pH 7.4; cyclic voltammetry data for MVCA-OH and p(MVCA-BAC); FTIR spectra of BAC, MVCA-OAcr and p(MVCA-BAC); cytotoxicity of MVCA-OH and p(MVCA-BAC) in M-HeLa, PC-3, WI-38, Chang liver cell lines; hemolytic activity of THPP@p(MVCA-BAC) and p(MVCA-BAC).

File 1 Additional experimental data.

## Data Availability

Data generated and analyzed during this study is available from the corresponding author upon reasonable request.
