# Supplementary material for: A redox-responsive viologen–cavitand nanocarrier for dual-function photodynamic therapy
Source: Beilstein J Nanotechnol. 2026 Aug 19;17:1142–58. doi: 10.3762/bjnano.17.78 (PMC13501230; doi:10.3762/bjnano.17.78)
Supplement: Supplementary file 1 [file Beilstein_J_Nanotechnol-17-1142-s001.pdf]

BEILSTEIN JOURNAL OF NANOTECHNOLOGY

Supporting Information

for

A redox-responsive viologen–cavitand nanocarrier for dual-function photodynamic therapy

Andrey A. Maslennikov, Alexey Yu. Usanyov, Amir A. Shamsutdinov, Anna P. Lyubina, Andrey A. Parfenov, Alexandra D. Voloshina, Irek R. Nizameev, Marsil K. Kadirov, Rezeda R. Fazleeva, Vitaly V. Yanilkin, Kseniya A. Zhdanova, Natal’ya A. Bragina, Albina Y. Ziganshina and Igor S. Antipin

Beilstein J. Nanotechnol. **2026,** *17,* 1142–1158. doi:10.3762/bjnano.17.78

Additional experimental data

License and Terms: This is a supporting information file under the terms of the Creative Commons Attribution License (https://creativecommons.org/licenses/by/4.0). Please note that the reuse, redistribution and reproduction in particular requires that the author(s) and source are credited and that individual graphics may be subject to special legal provisions.
The license is subject to the *Beilstein Journal of Nanotechnology* terms and conditions: (https://www.beilstein-journals.org/bjnano/terms)

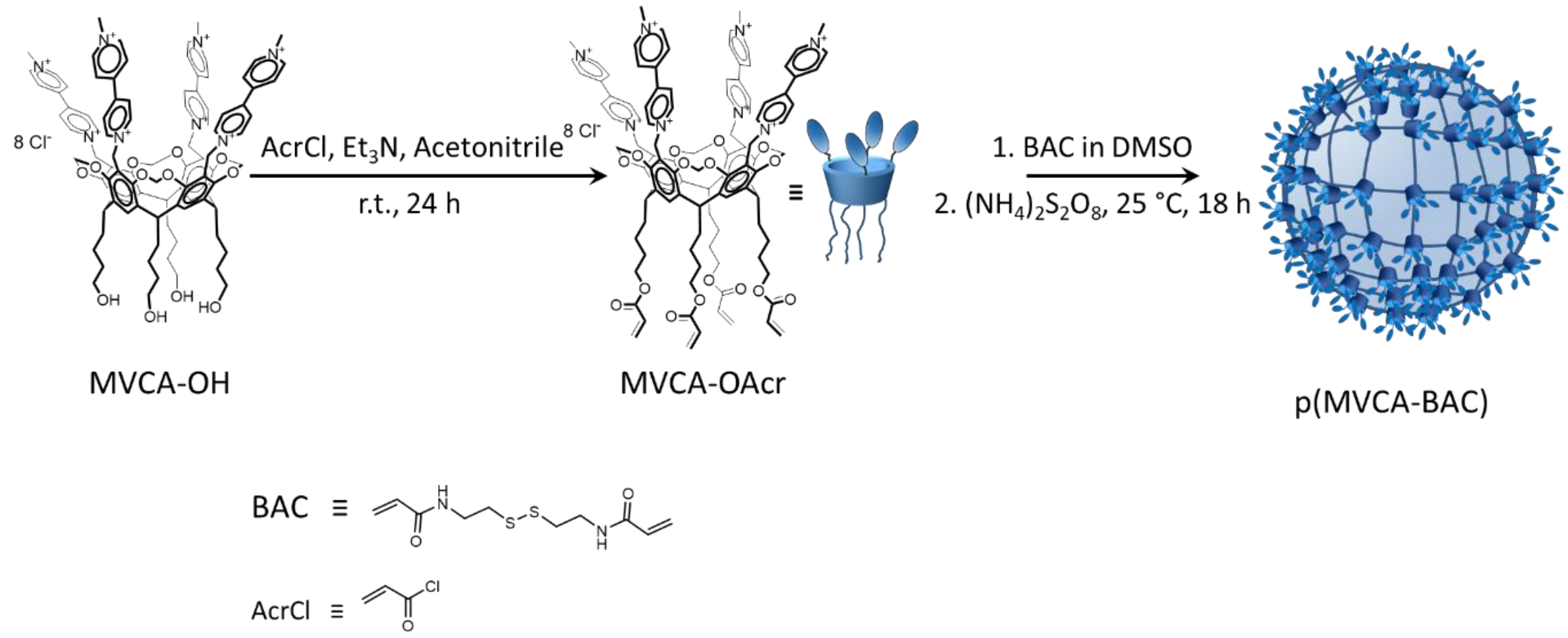

Scheme S1: Synthesis of p(MVCA-BAC).

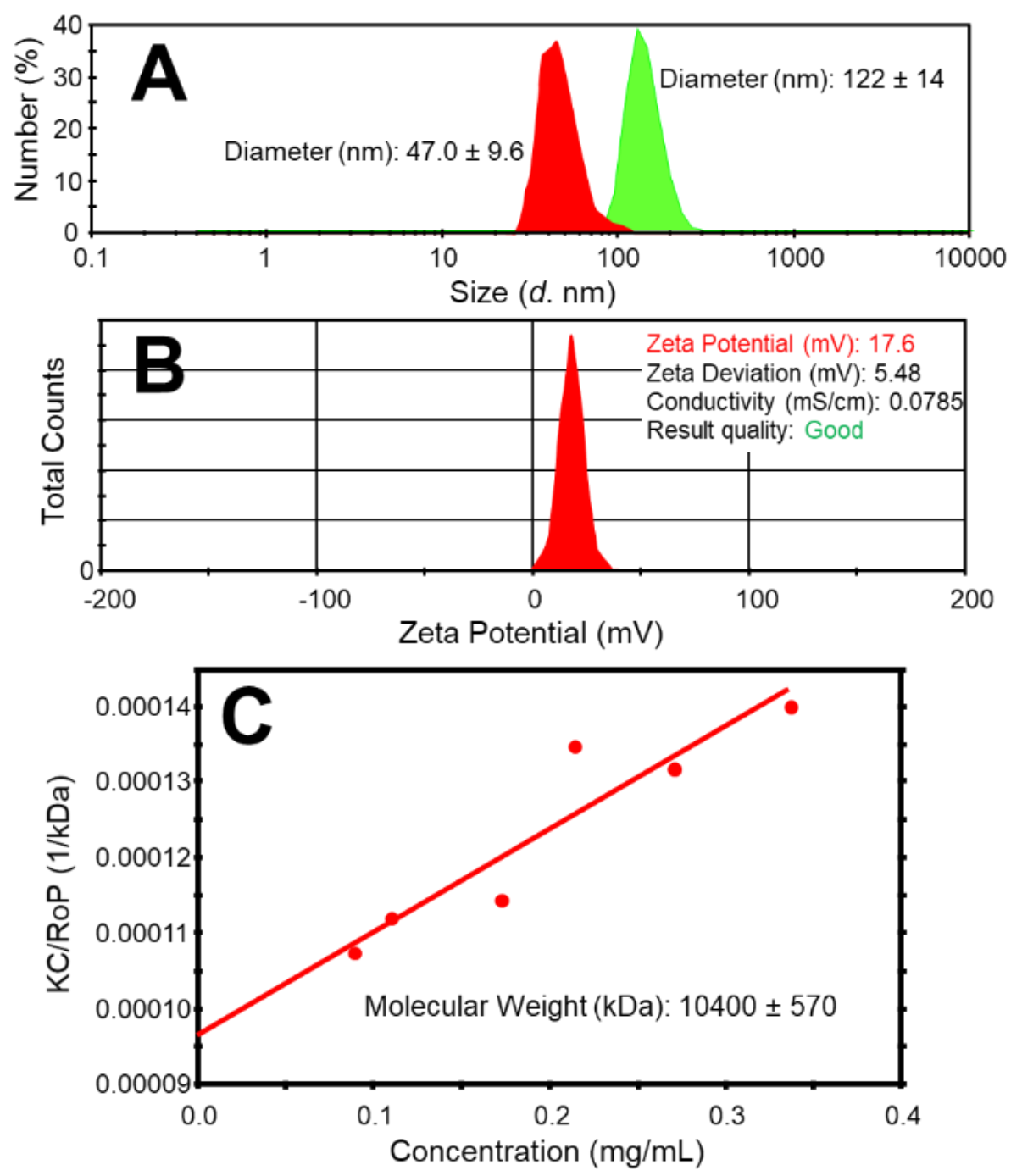

Figure S1: Data for p(MVCA-BAC): (A) Hydrodynamic size distribution, determined by DLS, before (red) and after (green) 1-hour incubation in 8 mM GSH; (B) Zeta potential distribution in aqueous media; (C) Debye plot for molecular weight estimation via SLS.

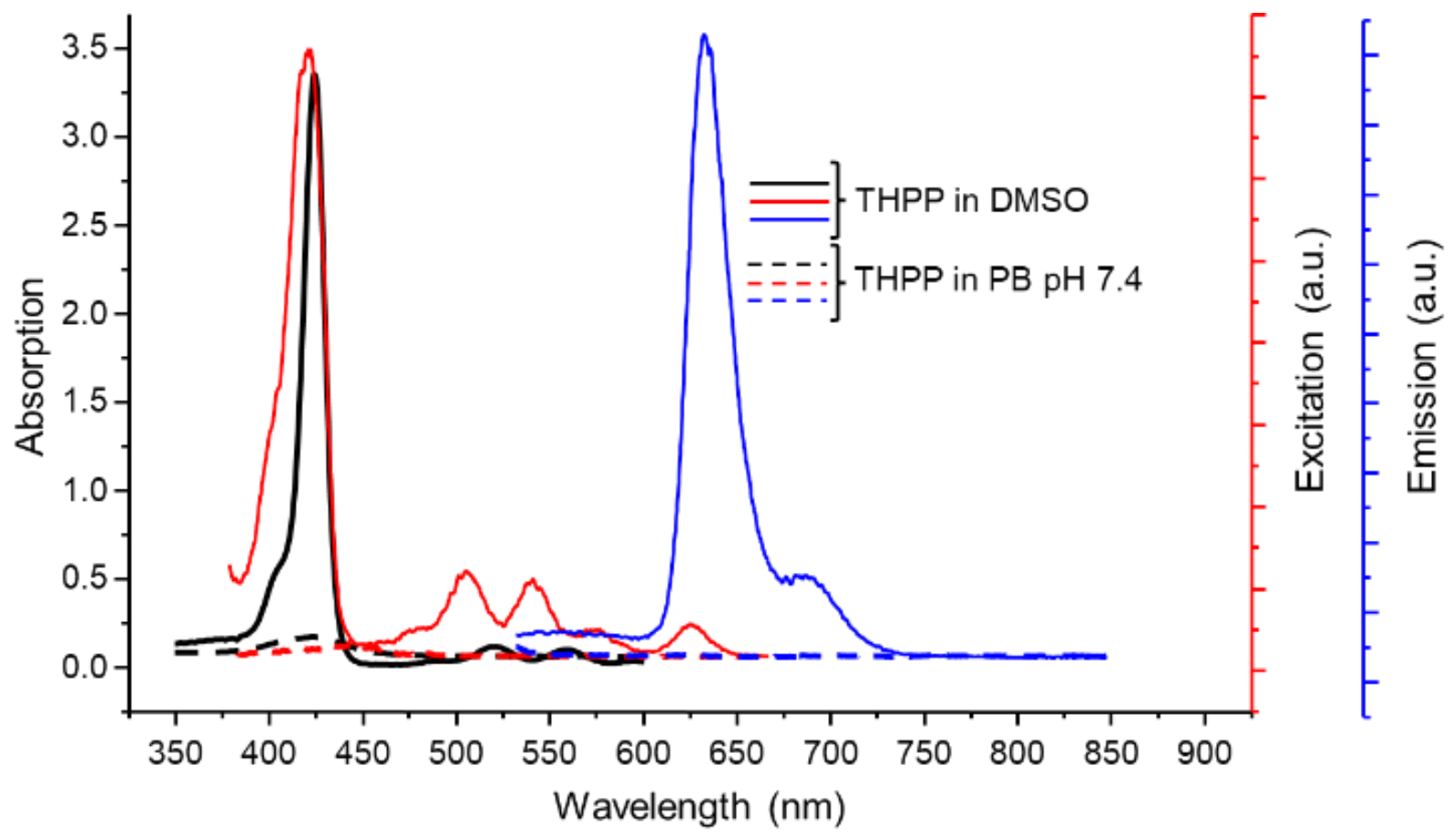

Figure S2: Optical spectra of free THPP in DMSO and PB pH 7.4: UV–Vis absorption (black), fluorescence excitation (red), and emission (blue) spectra *C*(THPP) = 3 μM at 37 °C.

Cyclic voltammetry (CV) analysis

CV of MVCA-OH and p(MVCA-BAC) (0.5 mM in 0.1 M NaCl, scan rate 100 mV/s) exhibit two reversible reduction–oxidation couples in the cathodic region, corresponding to the stepwise reduction of viologen units to the radical cation ($V^{+\bullet}$) and neutral form (V^0) (Figure S4, Table S2).

The peak currents of p(MVCA-BAC) are approximately 1.5-fold lower than those of MVCA, consistent with the reduced diffusion coefficient of the polymeric species relative to the molecule monomer.

Critically, the anodic oxidation peak at +0.60 V (vs SCE), assigned to the -OH group of the lower rim in MVCA, is absent in the polymer, confirming covalent incorporation of the monomer through the hydroxyl functionality during polymerization [1].

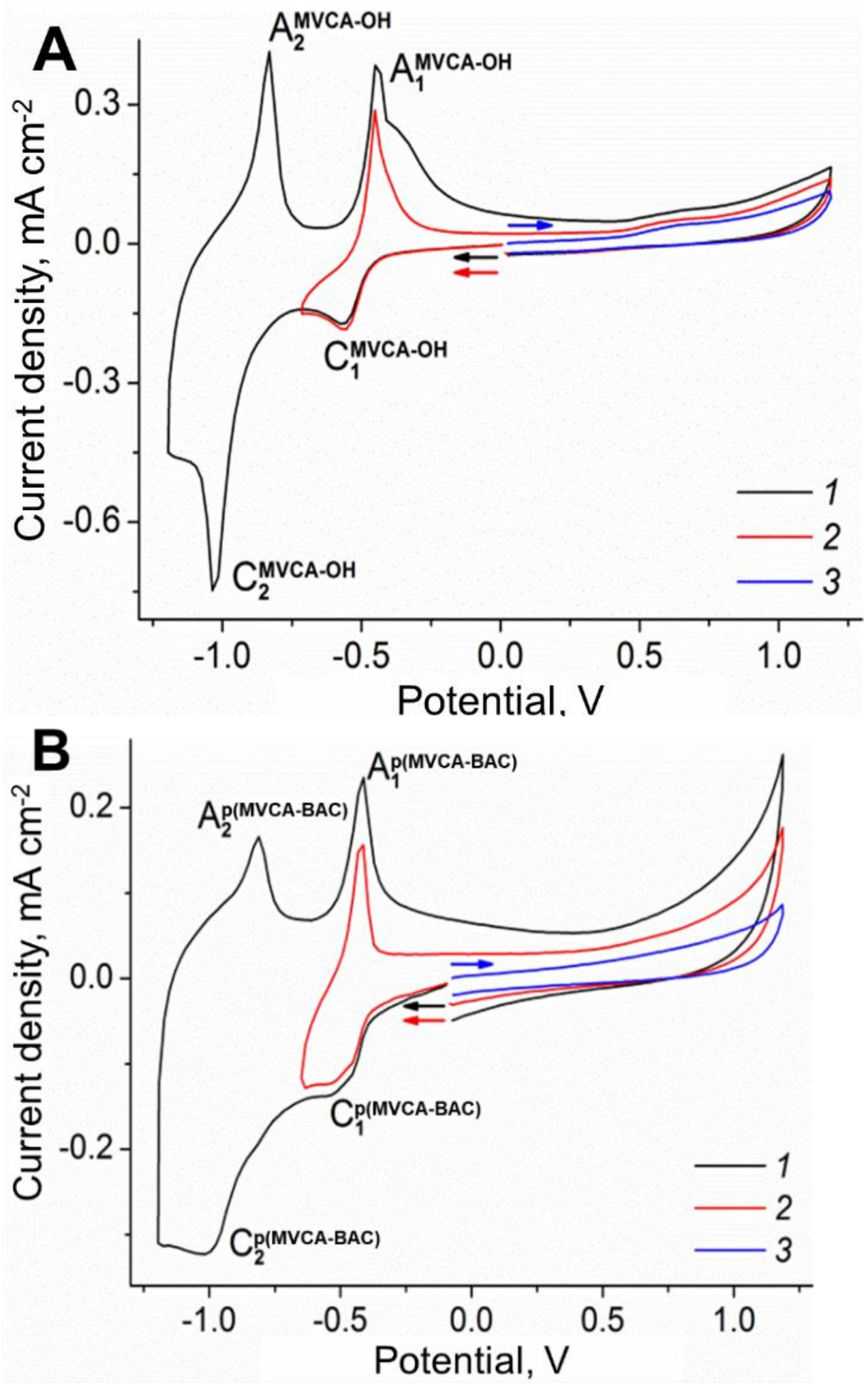

Figure S3: Cyclic voltammograms of (a) MVCA-OH and (b) p(MVCA-BAC) (0.5 mM) in aqueous 0.1 M NaCl (scan rate: 100 mV/s, vs SCE).

Table S1: Redox potentials (vs. SCE) of MVCA-OH and p(MVCA-NSS) (0.5 mM) in 0.1 M NaCl aqueous solution (scan rate: 100 mV/s); E_{C1} and E_{C2} denote cathodic peak potentials, E_{A1} and E_{A2} denote corresponding anodic peak potentials.

Compound	E_{C1}, V	E_{A1}, V	E_{C2}, V	E_{A2}, V
MVCA-OH	–0.56	–0.45	–1.03	–0.83
p(MVCA-BAC)	–0.53	–0.42	–1.01	–0.81

FTIR spectra

The FTIR spectrum of p(MVCA-BAC) exhibits characteristic bands of both MVCA-OAcr and BAC monomers (Figure S4). A broad absorption centered at 3252 cm^{-1} is assigned to ν(N-H) stretching vibrations of the amide groups in BAC, overlapping with overtones of the C-N bond of MVCA fragment. The peak at 1556 cm^{-1} corresponds to a combination of δ(N–H) bending and C–N stretching, confirming successful copolymerization between MVCA-OAcr and BAC.

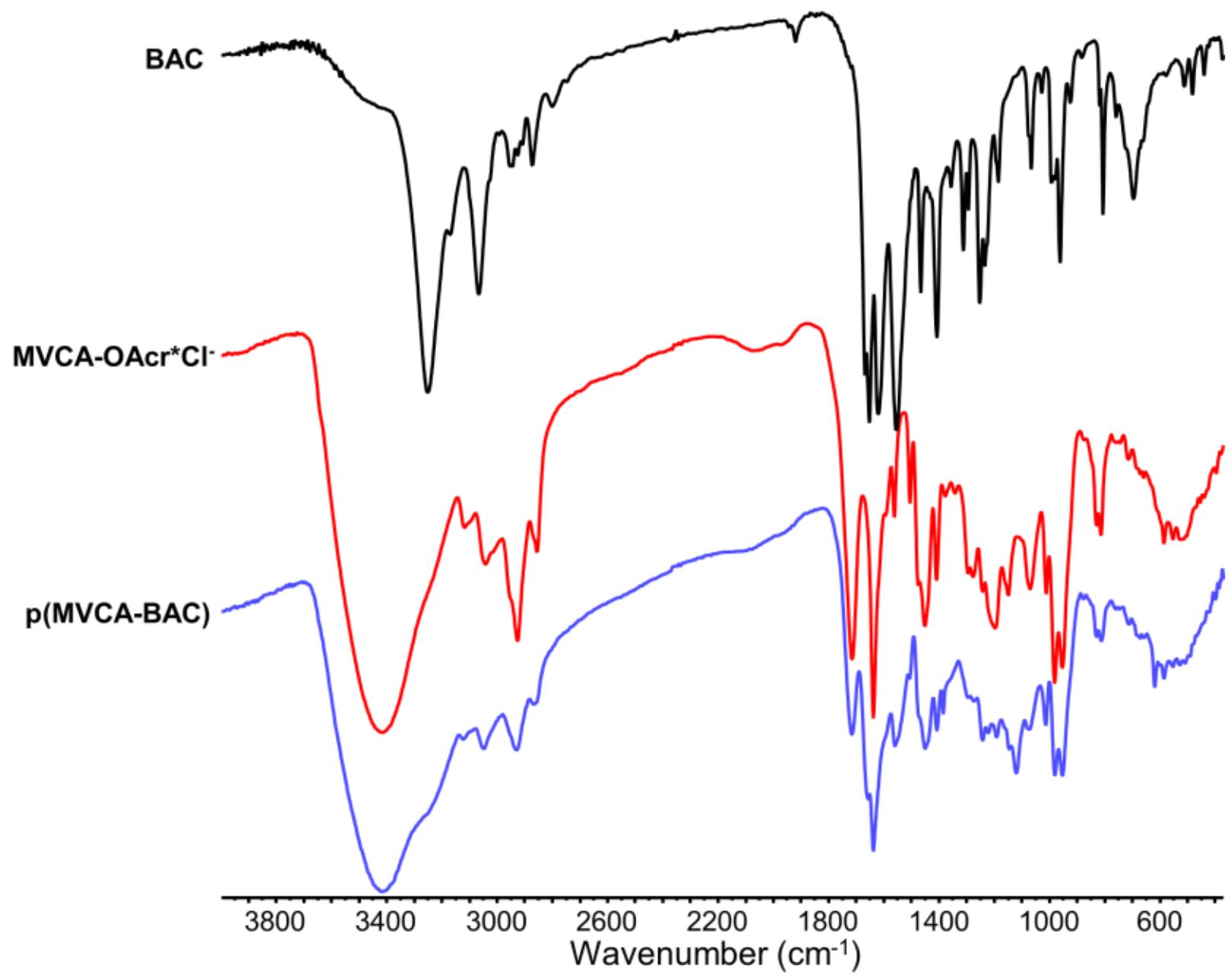

Figure S4: FTIR spectra of MVCA-OAcr, BAC and p(MVCA-BAC) in KBr.

Biological investigation

Table S2: Cytotoxicity of nanocarrier components in human cell lines, IC_{50} values (µM) of MVCA-OH and p(MVCA-BAC) after 24 h incubation in cancerous (M-HeLa, PC-3) and non-tumorigenic (WI-38, Chang liver) cell lines, data are mean ± SD ($n \geq 3$).

Compound	M-HeLa	PC-3	WI-38	Chang liver
MVCA-OH	247.9 ± 4.4	192 ± 0.6	166 ± 9.2	262 ± 18
p(MVCA-BAC)	258.3 ± 13	>250	238 ± 7.4	253 ± 6.5

Table S3: Hemolytic activity of THPP@p(MVCA-BAC) and empty p(MVCA-BAC) nanoparticles, data are mean ± SD ($n = 3$).

	C(MVCA), mM	Hemolysis, %	HC_{50}, mM (MVCA)
	0.05	6.9 ± 0.6	
	0.025	4.1 ± 0.3	
THHP@p(MVCA-BAC)	0.0125	5.8 ± 0.9	>0.05
	0.00625	5.6 ± 0.2	
	0.003125	0.3 ± 0.06	
	0.05	3.4 ± 1.0	
	0.025	3.2 ± 0.7	
p(MVCA-BAC)	0.0125	4.4 ± 0.7	>0.05
	0.00625	0.8 ± 0.2	
	0.003125	0.4 ± 0.1	

Reference

1. Zhang, Q.; Chen, T.; Jiang, R.; Jiang, F. Comparison of Electrocatalytic Activity of Pt1−xPdx/C Catalysts for Ethanol Electro-Oxidation in Acidic and Alkaline Media. *RSC Adv.* **2020**, *10*, 10134-10143.
